# Bispecific Antibody Targeting VEGF/TGF‐β Synergizes with Local Radiotherapy: Turning Tumors from Cold to Inflamed and Amplifying Abscopal Effects

**DOI:** 10.1002/advs.202501819

**Published:** 2025-06-05

**Authors:** Lijuan Lyu, Ming Yi, Ji Chen, Jing Zhang, Xiaobin Ma, Xiaojun Zhang, Liang Zeng, Yan Xue, Haimei Wen, Yujiao Deng, Pengfei Zhou, Kongming Wu, Huafeng Kang, Zhijun Dai

**Affiliations:** ^1^ The Comprehensive Breast Care Center The Second Affiliated Hospital of Xi'an Jiaotong University Xi'an 710004 China; ^2^ Department of Breast Surgery The First Affiliated Hospital College of Medicine Zhejiang University Hangzhou 310000 China; ^3^ Department of Radiation and Medical Oncology Hubei Key Laboratory of Tumor Biological Behacviors Hubei Cancer Clinical Study Center Zhongnan Hospital of Wuhan University Wuhan 430071 China; ^4^ Wuhan YZY Biopharma Co., Ltd Biolake, C2‐1, No.666 Gaoxin Road Wuhan 430075 China; ^5^ Cancer Center, Shanxi Bethune Hospital, Shanxi Academy of Medical Science, Tongji Shanxi Hospital Third Hospital of Shanxi Medical University Taiyuan 030032 China; ^6^ Cancer Center, Tongji Hospital of Tongji Medical College Huazhong University of Science and Technology 1095 Jiefang Avenue Wuhan 430030 China

**Keywords:** abscopal effects, bispecific antibody, radiotherapy, tumor microenvironment, TGF‐β, VEGF

## Abstract

Beyond inherently killing cancer cells by directly inducing double‐strand DNA breaks, local radiotherapy (RT) can exert immune‐priming effects and reprogram the tumor microenvironment (TME) from immune‐cold tumors into inflamed, or “hot” tumors. Nevertheless, this immunogenic antitumor response may be partially counterbalanced by the upregulation of vascular endothelial growth factor (VEGF) and transforming growth factor‐beta (TGF‐β) induced by RT. Y332D, a bispecific antibody that simultaneously blockades VEGF and TGF‐β, effectively synergized with RT, leading to a durable antitumor effect. Mechanistically, Y332D counteracted negative RT effects that are attributed to the upregulation of TGF‐β and VEGF‐mediated epithelial‐mesenchymal transition, extracellular matrix remodeling, aberrant angiogenesis, immunosuppression, and radioresistance, as well as further enhanced or complemented the positive effects of RT, such as cGAS‐STING activation, immunogenic cell death, enhanced antigen presentation, increased T cell infiltration, and antiangiogenic effects, thereby reprograming the TME from immune “cold” to inflamed state and forming an effective in‐situ vaccine that, beyond local tumor eradication, could potentiate antitumor immune response and regress preestablished abscopal metastases. Together, our results indicate that this combination strategy successfully overcame the negative effects caused by RT and augmented abscopal effects, extending the application of RT to the treatment of both local and metastatic disease.

## Graphical abstract

Y332D, a bispecific antibody that simultaneously blockades VEGF and TGF‐β, counteracted negative RT effects that are attributed to the upregulation of TGF‐β and VEGF, as well as further enhanced the immunostimulatory effects of RT, thereby reprograming the TME from immune “cold” to inflamed state and forming an effective in‐situ vaccine that eradicate local tumor and augment abscopal effects.

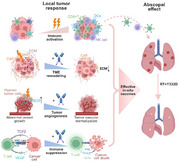

## 1 Introduction

Immunosuppressive pathways in the TME are inextricably linked to tumor progression. Mono‐therapeutics is prone to immune escape while combination therapeutics tends to cause high toxicity and side effects. Therefore, using bifunctional molecules to block two vital signal pathways simultaneously is becoming a new strategy for cancer therapies.^[^ ^1^ ^]^ Bispecific antibody (BsAb) is an artificial antibody that can specifically bind two antigens or antigenic epitopes at the same time.^[^ ^2^ ^]^ Compared with the combination of two mono‐functional antibodies, double‐blocking bifunctional antibodies are more likely to exert synergistic effects due to their special structure and higher targeting of tumor tissues.^[^ ^3^ ^]^ The remarkable observations in cancer progression are increased expressions of VEGF and TGF‐β in the tumor microenvironment (TME).^[^ ^4^ ^]^ Especially, a study has shown that blockade of TGF‐β induced tumor tissue hypoxia, which triggers cellular adaptation to resolve ischemia via the induction VEGF, and that TGF‐β blockade can be combined with VEGF inhibitors to further restrain the tumor vasculature‐mediated cancer progression.^[^ ^5^ ^]^ Meanwhile, VEGF inhibitors could induce TGF‐β up‐regulation due to hypoxia.^[^ ^6^ ^]^ Based on the complementary mechanism between VEGF and TGF‐β and their important roles on the TME, Wuhan YZY Biopharma has developed Y332D, a BsAb that simultaneously blockades mouse VEGF and TGF‐β, which is characterized by higher antigen affinity, greater stability, and lower immunogenicity, and that could inhibit tumor neovascularization, reduce tumor metastasis, and restore the TME from immunosuppressive to immune‐supportive status, thereby potentiating the efficacy of immunotherapy.^[^ ^7^ ^]^

Radiotherapy (RT) has become a mainstay of first‐line treatment in numerous solid tumors. Beyond its established role in inducing double‐strand DNA breaks to directly kill tumor cells, RT is now recognized for its profound immunostimulatory effects.^[^ ^8^ ^]^ Specifically, local RT can exert immune‐priming effects and transform the “cold” tumors defined by little T cell infiltration and poor immunogenicity into the “hot” tumors, marked by substantial T cell infiltration and high immunogenicity through several mechanisms. First, RT‐induced immunogenic cell death (ICD) of tumor cells releases plenty of neoantigens resulting from RT‐driven immunogenic mutations and damage‐associated molecular patterns (DAMPs), which serve as in‐situ vaccines that lead to dendritic cells (DCs) activation and effector T cell priming.^[^ ^9^, ^10^, ^11^ ^]^ Moreover, cytosolic DNA from damaged nuclei can be sensed by the cGAS‐STING pathway, leading to the induction of type I interferons (Type I IFNs) and the expression of other pro‐inflammatory genes along this pathway.^[^ ^12^, ^13^ ^]^ Type I IFNs mediate robust immunostimulatory effects upon binding to IFNAR1 on various immune cells.^[^ ^14^, ^15^ ^]^ Besides, RT can promote the secretion of chemokines necessary for T cell attraction, such as CCL5, CXCL9, and CXCL10, which facilitate the recruitment of CXCR3‐positive T cells into the TME.^[^ ^13^, ^16^ ^]^ Concurrently, RT increases major histocompatibility complex class I (MHC‐I) expression on the surface of tumor cells and antigen‐presenting cells (APCs) and alters the MHC‐I‐associated peptide profile, which facilitates cross‐presentation and restores immune recognition by cytotoxic CD8+ T cells (CTLs).^[^ ^9^, ^17^ ^]^ Additionally, RT inhibits ongoing angiogenesis and mainly impairs immature vessels, while more mature vessels show resistance to RT.^[^ ^18^ ^]^ Such evidence provides a strong rationale for the use of immunomodulators to boost the therapeutic value of RT. An increasing amount of preclinical and clinical data concerning the combination of RT with immunomodulators, particularly immune checkpoint inhibitors (ICIs), suggests that local RT synergistically with immunomodulators induces an “in‐situ tumor vaccines”, leading to systemic anti‐tumor immunity and tumor regression outside of the local radiation field, a phenomenon referred to as the “abscopal effect”.^[^ ^19^, ^20^ ^]^ Generally, the abscopal effect is so rare in solid tumors receiving RT alone that it has no broad application value seemingly. Whereas, with the advent of immunotherapy, it is now widely accepted that combining RT with immunomodulators provides an opportunity to boost abscopal response rates, extending the application of RT to the treatment of both local and metastatic disease.^[^ ^20^, ^21^ ^]^

Conversely, RT also exerts a negative effect on TME by promoting the secretion of immunosuppressive factors. Prior research has indicated an increase and activation of TGF‐β in irradiated tissues.^[^ ^22^, ^23^, ^24^, ^25^ ^]^ TGF‐β is a key contributor to the immunosuppressive TME and RT‐induced TGF‐β may hinder the development of anti‐tumor immune responses that can be elicited by RT. The biological functions of TGF‐β in TME are to inhibit the proliferation and differentiation of antitumor immune cells while promoting Tregs differentiation and M2 macrophage polarization.^[^ ^26^, ^27^, ^28^, ^29^ ^]^ As a key inducer of stromal activation in the TME, RT‐induced TGF‐β can activate tumor‐associated fibroblasts (CAFs) and lead to extracellular matrix (ECM) deposition, creating a dense barrier in the peritumoral region that hampered T cell infiltration.^[^ ^26^, ^30^ ^]^ Additionally, TGF‐β‐mediated epithelial‐mesenchymal transition (EMT) has been implicated in the accelerated progression of cancer metastasis following RT.^[^ ^31^ ^]^ Blocking TGF‐β has been demonstrated to prevent RT‐induced metastases in tumor‐bearing murine models.^[^ ^32^ ^]^ Moreover, TGF‐β can protect cancer cells from DNA damage by promoting DNA damage repair (DDR) mechanisms, thus decreasing radiosensitivity.^[^ ^33^, ^34^ ^]^ The blockade of TGF‐β signaling has been shown to increase radiosensitivity.^[^ ^35^, ^36^ ^]^ In parallel, RT can enhance the expression of VEGF in irradiated tumors by activating hypoxia‐inducible factor 1 (HIF‐1) signaling caused by RT‐induced hypoxia.^[^ ^37^, ^38^ ^]^ Moreover, elevated serum VEGF levels have been observed in certain patients with malignant tumors following RT.^[^ ^39^, ^40^ ^]^ Excessive levels of VEGF within the TME directly inhibit CTLs effector function and DCs maturation and antigen presentation, as well as also promote the recruitment and proliferation of immunosuppressive cells, including Treg cells, myeloid‐derived suppressor cells (MDSCs), and pro‐tumor, M2‐like macrophages.^[^ ^41^, ^42^, ^43^ ^]^ Besides, VEGF contributes to the formation of aberrant tumor vasculature that is structurally and functionally abnormal, resulting in an abnormal TME characterized by interstitial hypertension, hypoxia, and acidosis, which further fosters immunosuppression both locally and systemically.^[^ ^44^, ^45^ ^]^ Interstitial hypertension restricts immune cell infiltration into the tumor, and hypoxia and acidosis impair the activity of immune cells.^[^ ^45^ ^]^ Besides, hypoxia has long been known to render radiation less effective and vessel normalization could increase RT response and reduce tumor recurrence and metastasis.^[^ ^46^, ^47^ ^]^ VEGF also exerts pro‐metastatic effects by causing structural and biochemical abnormality in tumor blood and lymphatic vessels.^[^ ^48^ ^]^ Importantly, elevated VEGF and TGF‐β into the circulation can lead to systemic immunosuppressive, thereby impairing the abscopal effect typically induced by RT.^[^ ^49^ ^]^

In this study, we propose and test the hypothesis that Y332D may synergize with RT to eradicate immune “cold” tumors and amplify abscopal effects by reversing the negative effects of RT that are attributed to the upregulation of TGF‐β and VEGF and further enhancing or complementing its positive effects on the TME.

## 2 Results

### 2.1 RT Activated the cGAS‐STING Pathway and Triggered Type I Interferon Responses

**Figure 1:**
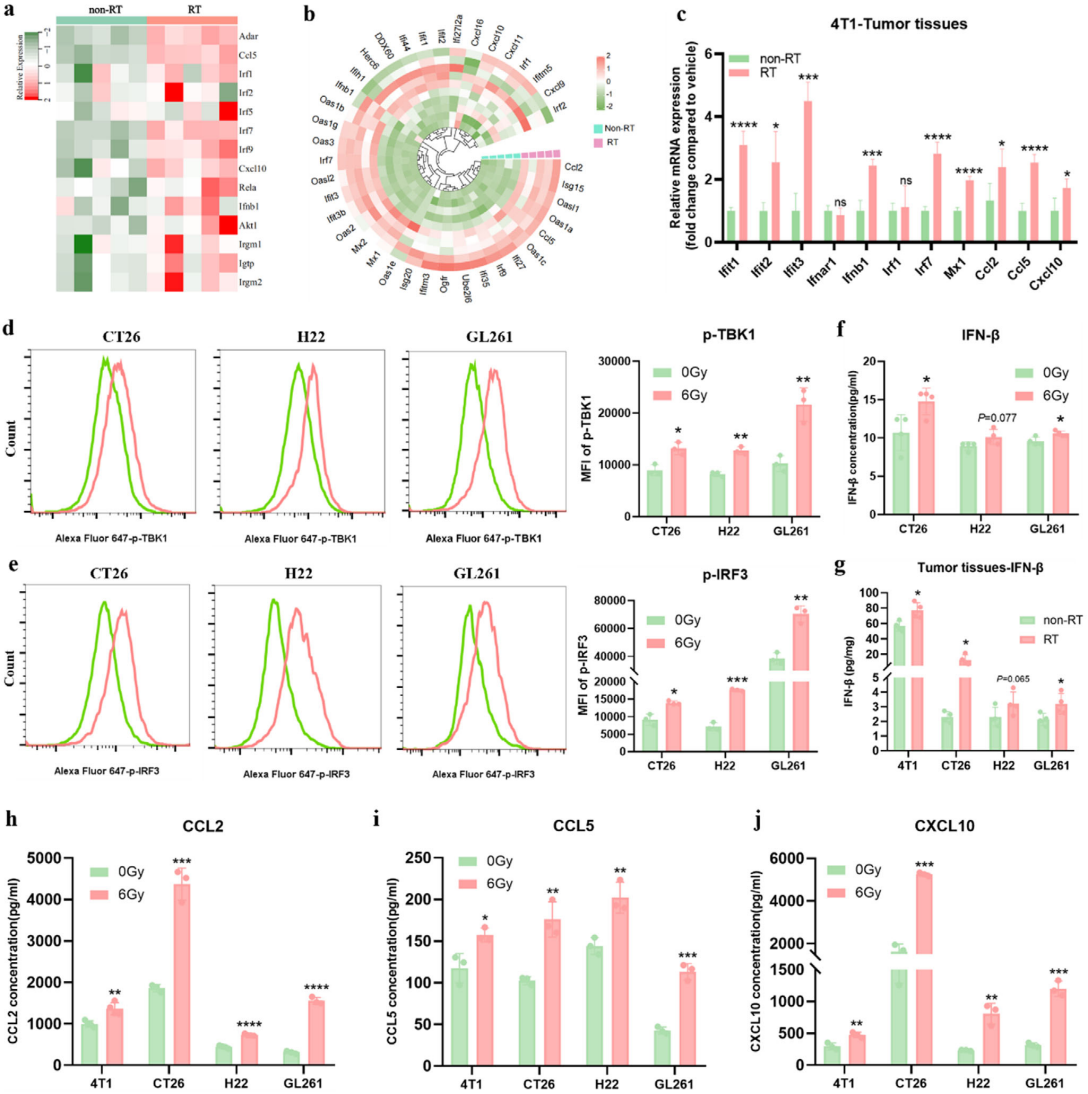
RT activated the cGAS‐STING pathway and induced a type I IFN response. a, b) Heatmap showing the expression levels of STING signaling pathway genes and IFN‐I‐stimulated genes (ISGs) detected by RNA‐seq 3 days post‐irradiated (6 Gy*3) or non‐irradiated 4T1 tumor tissues (*n =* 5), respectively. c) The mRNA expressions of a panel of 11 ISGs in 4T1 tumor tissues 3 days after irradiation (6Gy*3) were confirmed by qRT‐PCR (*n =* 3). d, e) phosflow cytometry assays showed the phosphorylated levels of TBK‐1 and IRF‐3 in CT26, H22, and GL261 cells at 24 h post‐irradiation (*n =* 3). The mean fluorescence intensities (MFIs) of phosphorylated‐TBK1 (p‐TBK1) and phosphorylated‐IRF3 (p‐IRF3) were quantified. f) The concentration of IFN‐β in supernatants of CT26, H22, and GL261 cells were measured 24 h after in vitro irradiation versus non‐irradiation (*n =* 3). g) The IFN‐β level in tumor tissue homogenates of 4T1, CT26, H22, and GL261 tumors was assessed at 3 days post‐RT (6Gy*3) (*n =* 4). h‐j) The chemokines of CCL2, CCL5, and CXCL10 in the supernatants of 4T1, CT26, H22, and GL261 cells were detected 48 h after irradiation or non‐irradiation (*n =* 3). All data are mean±SD. Statistical analyses were conducted using Student's *t*‐test. Significance is indicated as: *p < 0.05, **p < 0.01, ***p < 0.001, and ****p < 0.0001; ns: not significant.

To investigate whether RT activates the cGAS‐STING pathway and induces type I interferon responses in vivo, gene transcripts induced by RT in 4T1 tumors were analyzed 3 days after irradiation of 6Gy × 3, revealing the upregulation of STING signaling pathway genes and IFN‐I stimulated genes (ISGs) by 6Gy × 3 (**Figure** **1**a,b). Subsequent qRT‐PCR validation with a panel of 11 ISGs in vivo and in vitro confirmed these findings. We observed significant upregulation of the transcriptional levels of Ifnb1, Ifit1, Ifit2, Ifit3, Irf7, and Mx1 in irradiated 4T1 tumor tissues (Figure 1c) and irradiated CT26, H22, and GL261 carcinoma cells (Figure S1a, Supporting Information). Interestingly, in vitro, irradiation of 4T1 carcinoma cells in the absence of the tumor stroma showed upregulation of ISGs, except Ifnb1 (Figure S1a, Supporting Information), suggesting that the cGAS‐STING pathway may be variant in 4T1 cells and that tumor stromal cells play a critical role in RT‐activated immune responses. Subsequently, we detected the phosphorylation levels of TANK‐binding kinase 1 (TBK‐1) and interferon regulate factor 3 (IRF‐3) in CT26, H22, and GL261 cells using phosflow, and the IFN‐β level in tumor cell supernatants and tumor tissues homogenates by ELISA. Notably, irradiated tumor cells, including CT26, H22, and GL261 cells, exhibited significantly increased phosphorylation levels of TBK‐1 and IRF‐3 (Figure 1d,e). Meanwhile, we observed a marked elevation of IFN‐β cytokine in irradiated CT26 and GL261 cells and irradiated 4T1, CT26, and GL261 tumor tissues while a slight increase of IFN‐β cytokine in H22 cells and tumors following RT (Figure 1f,g). Additionally, IFN‐inducible chemokines CCL2, CCL5, and CXCL10 were found to be upregulated at both mRNA and protein levels in multiple cancer cells post‐irradiation (Figures 1h–j and S1a, Supporting Information).

### 2.2 RT‐induced ICD in Tumor Cells and Upregulated MHC‐I on the Tumor Cells Surface

**Figure 2:**
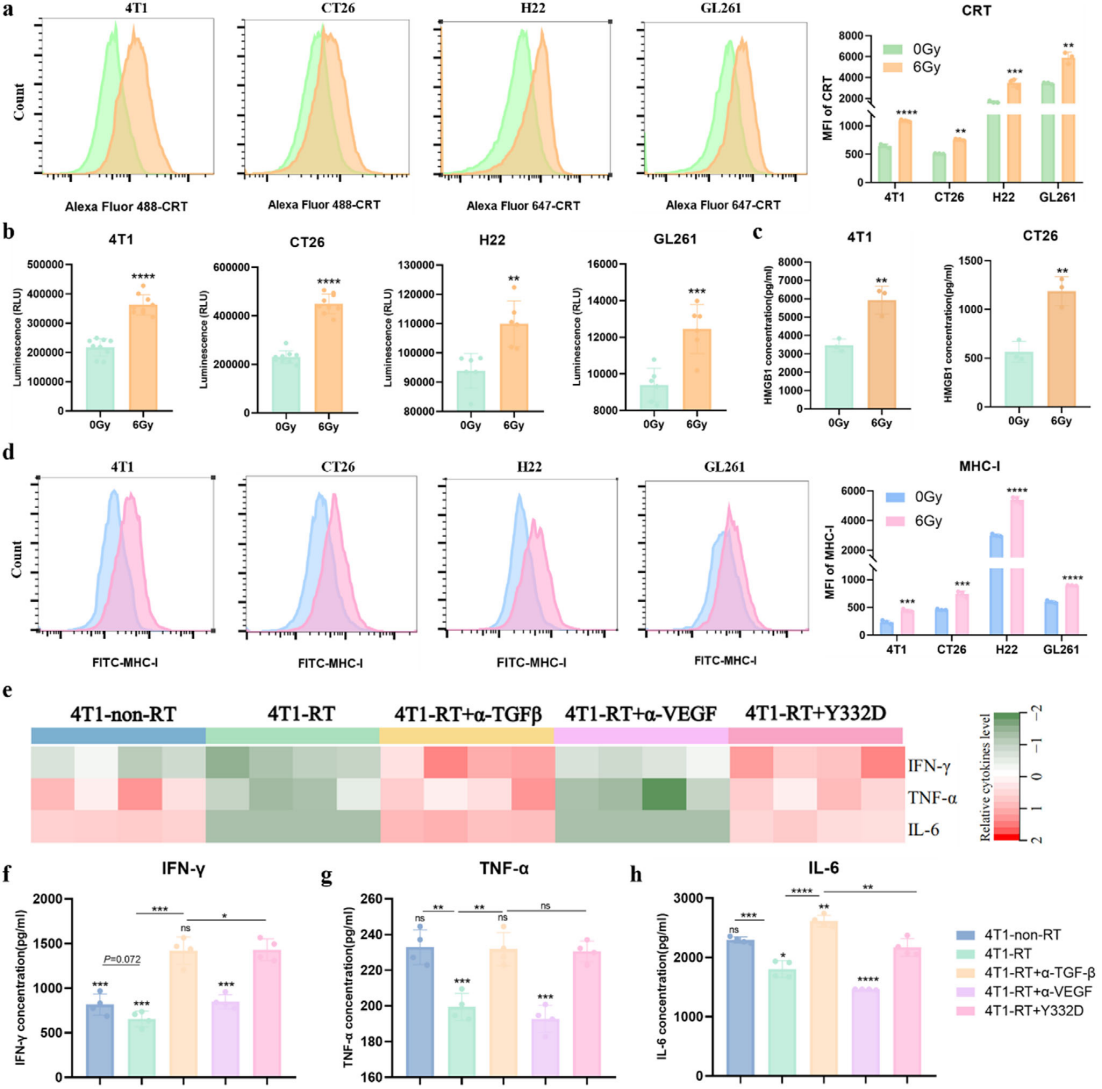
RT induced immunogenic cell death and increased MHC‐I expression on the surface of tumor cells. a,d) The expressions of calreticulin (CRT) and MHC‐I on the surface of 4T1, CT26, H22, and GL261 cells were detected by flow cytometry 24 h following irradiation or non‐irradiation (*n =* 3). b) ATP release from 4T1, CT26, H22, and GL261 cells was quantified 10 h post‐ irradiation or non‐irradiation (*n =* 6). c) The concentration of HMGB1 released into culture supernatants by 4T1 and CT26 cells was measured 48 h after irradiation or non‐irradiation (*n =* 3). e‐h) Cytokine levels in the supernatants of 4T1‐Fluc tumor cells and splenocytes co‐culture for 72 h were assessed (*n =* 4). The heatmap shows the relative level of cytokines, while the dot plots present the quantitative values. All data are mean±SD. Statistical analyses were conducted using Student's *t*‐test. Significance is indicated as: *p < 0.05, **p < 0.01, ***p < 0.001, and ****p < 0.0001; ns: not significant.

ICD is an inflammatory form of cell death that is implicated in the generation of antitumor adaptive immunity. During ICD, dying cells express and release of DAMPs that act through a diversity of mechanisms to increase antigen cross‐presentation and provide an inflammatory context for generating antigen‐specific T‐cell responses.^[^ ^50^ ^]^ We next investigated if RT‐mediated cell death was associated with several common and widely accepted biochemical hallmarks of ICD, including adenosine triphosphate (ATP) actively secreted, calreticulin (CRT) cell surface exposure and high mobility group protein B1 (HMGB1) passively released. Our findings revealed that RT significantly increased CRT exposure on the cell membranes compared to non‐irradiated tumor cells (**Figure** **2**a). Concurrently, irradiated 4T1, CT26, H22, and GL261 tumor cells significantly increased ATP secretion into the culture medium (Figure 2b). Besides, the extracellular HMGB1 levels were markedly elevated in 4T1 and GL261 cells upon exposure to RT (Figure 2c). Especially, RT‐upregulated MHC‐I expression on multiple tumor cell surfaces, including those of 4T1, CT26, H22, and GL261 cells (Figure 2d). To mimic the ICD induced by RT and tumor‐specific cytotoxicity triggered by ICD with or without Y332D in vitro, we co‐cultured 4T1‐Fluc cells (target cells), irradiated or non‐irradiated 4T1 cells, and splenocytes cells from BALB/c mouse (effector cells) (Figure S1b, Supporting Information). Compared to the non‐irradiated 4T1 cells co‐culture system, the irradiated 4T1 cells triggered a tumor‐specific cytotoxicity effect, which was further augmented by simultaneous blockade of TGF‐β and VEGF with Y332D (Figure S1c, Supporting Information). Furthermore, the secretion of pro‐inflammatory cytokines, including IFN‐γ, TNF‐α, and IL‐6, was attenuated in irradiated 4T1 cells co‐culture supernatants relative to non‐irradiated 4T1 cells co‐culture, potentially due to the RT‐induced promotion of TGF‐β secretion by tumor cells (Figure 2e–h). The blockade of TGF‐β using α‐TGF‐β or Y332D reversed and even further enhanced the secretion of pro‐inflammatory cytokines in the irradiated 4T1 cells co‐culture system (Figure 2e–h).

### 2.3 RT upregulated TGF‐β and VEGF in tumors, while Y332D could improve the radiosensitivity of tumor cells by blocking TGF‐β and VEGF

**Figure 3:**
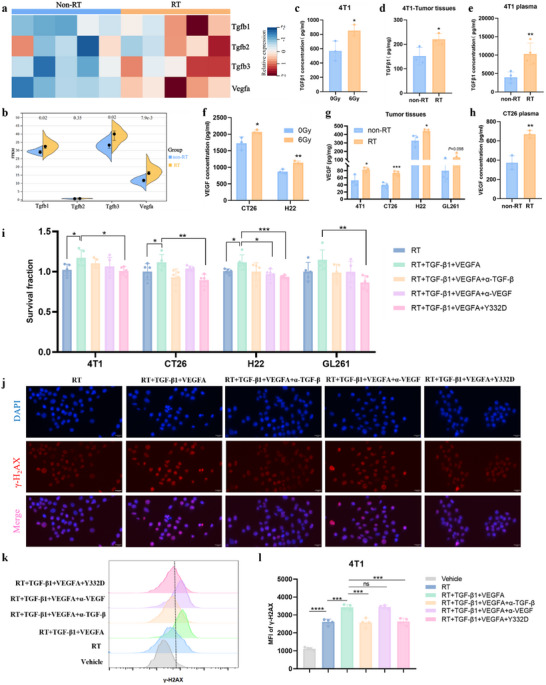
RT upregulated TGF‐β and VEGF, while Y332D could improve the radiosensitivity of tumor cells by blocking TGF‐β and VEGF. a, b) Heatmap and violin plot depicting TGF‐β1, TGF‐β2, TGF‐β3, and VEGFA expression patterns in 4T1 tumors at 3 days after irradiation (6Gy*3) and non‐irradiationdetected by RNA‐seq (*n =* 5). c) The concentration of TGF‐β1 in the 4T1 cell supernatants was measured 48 h after irradiation or non‐irradiation by ELISA (*n =* 3). d) The TGF‐β1 level in 4T1 tumor tissue homogenates at 3 days after RT (6 Gy*3) quantified by ELISA (*n =* 3). e) The TGF‐β1 concentration in the plasma of BALB/c mouse bearing 4T1 mammary carcinoma was measured 3 days after irradiation (6Gy*3) and non‐irradiation by ELISA (*n =* 4). f) The VEGFA concentration in the supernatants of CT26 and H22 cells was measured 48 h post‐irradiation or non‐irradiation using ELISA (*n =* 3). g) The VEGFA level in tumor tissue homogenates of 4T1, CT26, H22, and GL261 tumors at 3 days after RT (6 Gy*3) quantified by ELISA (*n =* 3 for 4T1 tumors and *n =* 4 for others). h) The VEGFA concentration in the plasma of BALB/c mouse bearing CT26 colon carcinoma was detected 48 h post‐irradiation (6Gy*3) and non‐irradiation detected by ELISA (*n =* 3). i) 4T1, CT26, H22, and GL261 cells pretreatment with exogenous TGF‐β1, VEGFA, and antibodies (10^6^ pm) for 24 h before irradiation, and the cell viability was evaluated by CCK‐8 24 h post‐irradiation (*n =* 5). (j) 4T1 cells pretreatment with exogenous TGF‐β1, VEGFA, and antibodies (10^6^ pm) for 24 h before irradiation, and the γ‐H2AX level in 4T1 cells was detected by IF staining 5 h post‐irradiation. (k, l) 4T1 cells pretreatment with exogenous TGF‐β1, VEGFA, and antibodies (10^6^ pm) for 24 h before irradiation, and the γ‐H2AX level in 4T1 cells was measured by phosflow cytometry 4 h post‐irradiation (*n =* 4). The size of the scale bar in immunofluorescence images refers to 10 µm. All data are mean±SD. Statistical analyses were conducted using Student's *t*‐test. Significance is indicated as: *p < 0.05, **p < 0.01, ***p < 0.001, and ****p < 0.0001; ns: not significant.

To determine whether RT induces the expression of TGF‐β and VEGF, we analyzed the transcription level of Tgfb1, Tgfb2, Tgfb3, and Vegfa using RNA‐seq data of irradiated versus non‐irradiated 4T1 tumor tissues. Significant upregulation of Tgfb1, Tgfb3, and Vegfa was observed in 4T1 tumor tissues post‐irradiation (**Figure** **3**a,b). We further validated the mRNA expression changes of Tgfb1 and Vegfa in various tumor cells after irradiation using qRT‐PCR. The results showed that RT promoted Tgfb1 mRNA expression in 4T1, CT26, H22, and GL261 tumor cells (Figure S2a, Supporting Information), which was similar to TGF‐β1 levels in the supernatants of 4T1, CT26, H22, and GL261 cells measured by ELISA (Figures 3c and S2b, Supporting Information). In vivo, we detected clearly elevated TGF‐β1 levels in both irradiated 4T1 tumor tissue and the plasma of BALB/c mouse bearing irradiated 4T1 mammary carcinoma (Figure 3d,e) while slightly increased TGF‐β1 levels in irradiated CT26, H22, and GL261 tumor tissues (Figure S2c, Supporting Information). Given the important role of TGF‐β1 in EMT progression,^[^ ^32^ ^]^ we detected the mRNA expression of EMT‐related genes (E‐cadherin, N‐cadherin, and Vimentin) in radiated versus non‐radiated tumor cells by qRT‐PCR and found that RT upregulated N‐cadherin and Vimentin mRNA of 4T1, CT26, H22, and GL261 tumor cells (Figure S2a, Supporting Information). Additionally, RT promoted Vegfa mRNA expression in CT26 and H22 cells (Figure S2a, Supporting Information), which was further confirmed by ELISA in the supernatants of these cells (Figure 3f). In vivo, increased VEGF level was detected in irradiated 4T1, CT26, H22, and GL261 tumor tissues (Figure 3g) and the plasma of BALB/c mouse bearing irradiated CT26 colon carcinoma (Figure 3h).

TGF‐β1 and VEGF have been reported to promote tumor radioresistance.^[^ ^35^, ^51^ ^]^ Therefore, we investigated the radiosensitivity of multiple tumor cells following preconditioning with exogenous TGF‐β1 and VEGFA or simultaneous blocking TGF‐β1 and VEGFA with Y332D. The CCK8 assays showed exogenous TGF‐β1 and VEGFA rendered tumor cells resistant to RT, while the blockade of TGF‐β1 and VEGFA with Y332D significantly inhibited survival fractions and proliferation of 4T1, CT26, H22, and GL261 tumor cells under irradiation (Figure 3i). DNA damage is an important component of radiation‐induced cytotoxicity, and TGF‐β1 has been reported to play a role in mediating DDR.^[^ ^35^ ^]^ To determine whether the blockade of TGF‐β1 could augment radiosensitivity through mediating DDR, we detected phosphorylated‐H2AX (γ‐H2AX), a key player in DDR, using immunofluorescence (IF) and phosflow. As depicted in Figure 3j, the formation of γ‐H2AX foci was notably enhanced in irradiated 4T1 cells pretreated with exogenous TGF‐β1, suggesting that exogenous TGF‐β1 facilitated DNA damage repair in tumor cells, thereby contributing to radioresistance. In line with the observed increase in radiosensitivity, the formation of RT‐induced γ‐H2AX foci in 4T1 cells was significantly diminished by α‐TGF‐β or Y332D‐mediated blockade of TGF‐β1 pretreatment, indicating that the enhancement of RT‐induced γ‐H2AX foci by TGF‐β1 could be reversed by α‐TGF‐β or Y332D and that Y332D enhances radiosensitivity by inhibiting the DNA damage repair effects of TGF‐β1 (Figure 3j). Consistent results were obtained using phosflow in 4T1 cells (Figure 3k,l).

### 2.4 Y332D Enhanced the Antitumor Effects of RT in Multiple Murine Tumor Models and Suppressed Spontaneous Lung Metastasis in an Orthotopic Breast Cancer Model

**Figure 4:**
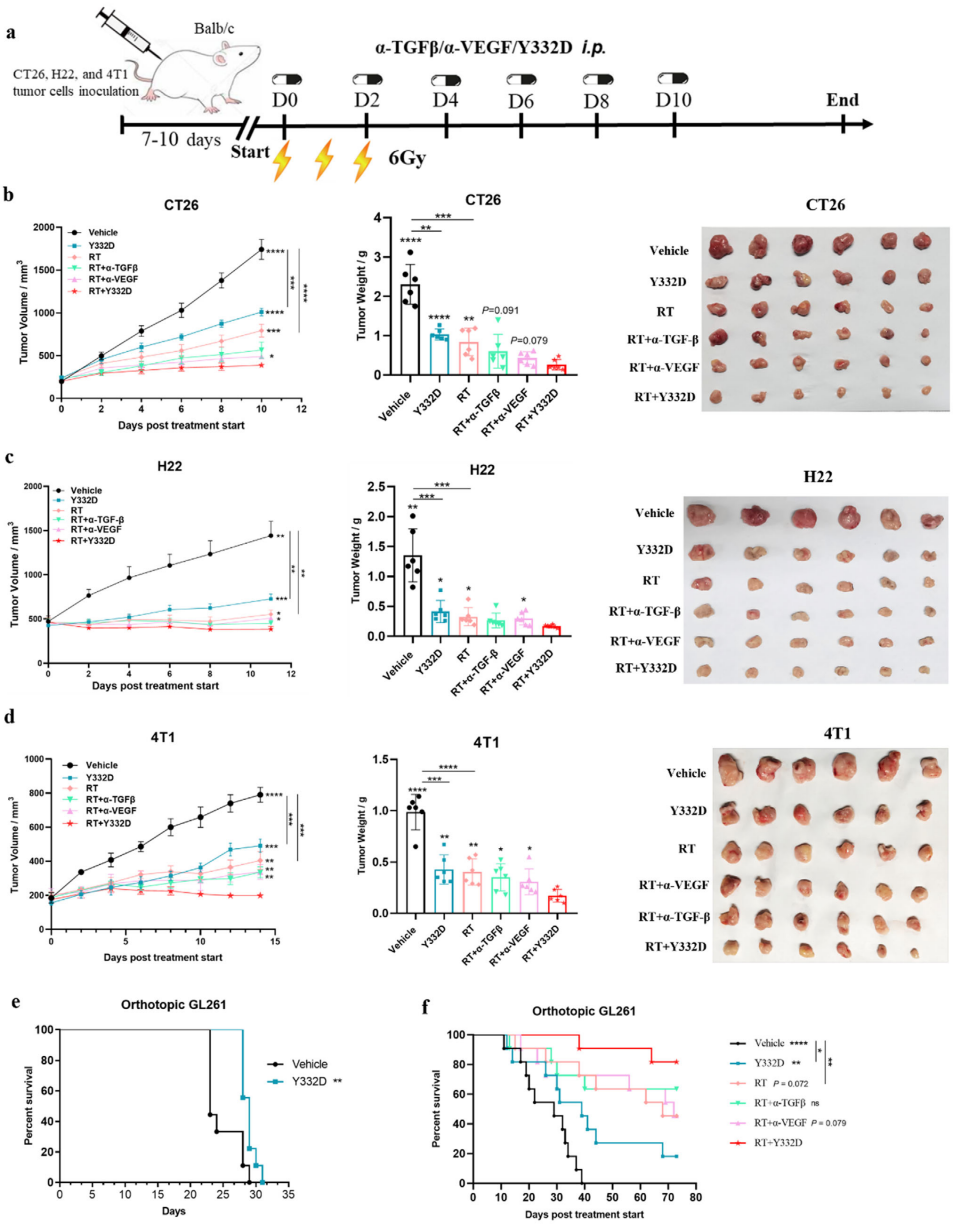
Y332D potentiated the antitumor efficacy of RT in four murine tumor models. a) Schematic of treatment regimens for CT26 and H22 subcutaneous tumor models, and 4T1 orthotopic tumor model. b‐d) Tumor growth curves, tumor weights, and photographic documentation of tumors from CT26, H22, and 4T1 tumor‐bearing mice that received RT and/or antibodies treatment (*n =* 6). Tumor volumes and weights are depicted as mean±SEM. e, f) RT plus Y332D therapy prolonged the survival of C57bl/6J mouse bearing orthotopic GL261 glioblastoma. Kaplan‐Meier survival curves for the orthotopic GL261 glioblastoma model by treatment group, (e) Y332D only (*n =* 9), (f) RT combined with Y332D (*n =* 11). Statistical analyses were conducted using Student's *t*‐test (CT26, H22, and 4T1 models) and Log‐rank test (for survival analysis). Statistical significance compared to the RT plus Y332D group is indicated as follows: *p < 0.05, **p < 0.01, ***p < 0.001, and ****p < 0.0001; ns: not significant.

We subsequently evaluated the synergistic antitumor activity of RT combined with Y332D across several murine tumor models with low immunogenicity, including CT26 colon carcinoma, H22 hepatocarcinoma, and 4T1 orthotopic breast cancer syngeneic models. The treatment schedule is depicted in **Figure** **4**a. Our findings indicated that both Y332D and RT monotherapies significantly delayed tumor growth and reduced tumor burden compared to the vehicle and that RT plus Y332D further suppressed tumor growth and diminished tumor burden relative to either monotherapy in these tumor models (Figure 4b–d). Notably, the combination of RT with Y332D outperformed RT with α‐VEGF in inhibiting tumor growth across the CT26, H22, and 4T1 tumor models (Figure 4b‐‐d). Concurrently, the combination of RT with Y332D was more efficacious in suppressing tumor growth and reducing tumor burden than RT with α‐TGF‐β in the 4T1 tumor model (Figure 4d). Furthermore, in this 4T1 orthotopic breast cancer model, Y332D more effectively decreased the incidence of spontaneous lung metastases than the vehicle (Figure S3a, Supporting Information). RT plus Y332D treatment further reduced the number of metastatic foci, which are slightly superior to RT plus α‐VEGF and similar to RT plus α‐TGF‐β (Figure S3a, Supporting Information).

Additionally, we investigated the impact of the combination therapy on survival in an orthotopic GL261 glioblastoma (GBM) mouse model. Orthotopic GL261 tumors growing in the brains of syngeneic C57BL/6 mice demonstrate limited tumor‐infiltrating lymphocytes (TILs).^[^ ^52^ ^]^ In this orthotopic GBM model (the treatment schedule is shown in Figure S3b, Supporting Information), Y332D monotherapy extended median survival (29 days) relative to vehicle (23 days) (Figure 4e). In a separate experiment (the treatment schedule is shown in Figure S3c, Supporting Information), Y332D monotherapy prolonged median survival (39 days) and increased complete tumor regressions (2 of 11 mice) relative to vehicle (29 days, 0 of 11 mice) (Figure 4f). RT monotherapy significantly extended median survival (68 days) and increased tumor regressions (5 of 11 mice) relative to vehicle (29 days, 0 of 10 mice) (Figure 4f). Meanwhile, RT plus Y332D treatment further slightly prolonged survival and increased tumor regressions (9 of 11 mice) to the end of the study relative to RT (p = 0.072, 5 of 11 mice) alone and RT plus α‐VEGF ((p = 0.079, 5 of 11 mice) (Figure 4f).

### 2.5 Y332D Counteracted RT‐induced VEGF and TGF‐β Upregulation, EMT Promotion, and CAF Activation, and Potentiated the Inhibition of Angiogenesis by RT

**Figure 5:**
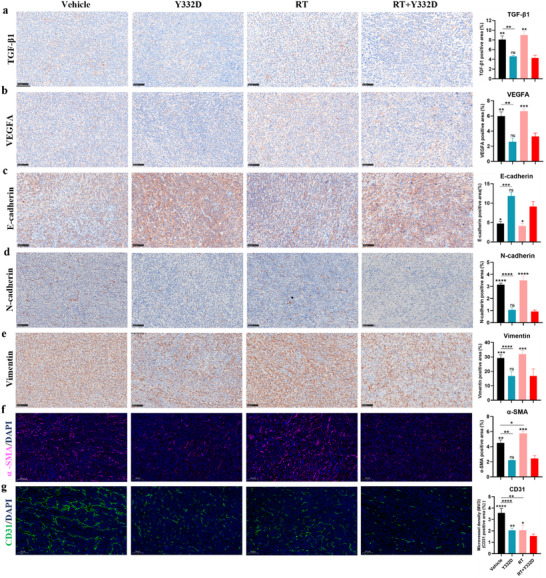
Impact of RT combined with Y332D on the TGF‐β signaling, VEGF pathway, epithelial‐mesenchymal transition (EMT), carcinoma‐associated fibroblasts (CAFs) activity, and tumor angiogenesis in the 4T1 tumor model. a–e) Representative IHC images and quantification of TGF‐β1 staining(a), VEGFA staining(b), and EMT‐related markers, including E‐cadherin(c), Vimentin(d), and N‐cadherin (e) staining, are presented (*n =* 5). f, g) Representative IF images and quantification of α‐SMA staining (f), a marker of CAF activation, and CD31 staining (g), a tumor vascular endothelial marker, are depicted (*n =* 5). The scale bar in IHC and IF images is 100 µm. All data are mean±SEM. Statistical analyses were conducted using Student's *t*‐test. Statistical significance compared to the RT combined with the Y332D group is indicated as follows: *p < 0.05, **p < 0.01, ***p < 0.001, and ****p < 0.0001; ns: not significant.

Given that RT has been shown to enhance the expression of VEGFA, TGF‐β1, and EMT‐related genes in vitro, we sought to determine whether Y332D could counteract RT‐induced upregulation of VEGFA and TGF‐β1, as well as the promotion of EMT in 4T1 tumor tissues via Immunohistochemistry (IHC) staining. Our findings revealed that RT slightly increased the expression of VEGFA and TGF‐β1 versus vehicle, and Y332D effectively reversed these RT‐induced increases in VEGF and TGF‐β expression (**Figure** **5**a,b). Similarly, RT slightly upregulated the expression of N‐cadherin and Vimentin while downregulated E‐cadherin compared to the vehicle, and Y332D counteracted the EMT phenotype caused by RT (Figure 5c–e). Simultaneously, Y332D significantly reduced stromal α‐SMA expression versus vehicle. Although RT significantly increased α‐SMA levels, Y332D was capable of reversing the RT‐induced α‐SMA (Figure 5f). Besides, Intratumoral microvessel density (MVD), a surrogate measure of angiogenesis in tumor models, was evaluated using CD31 staining. Relative to intratumoral MVD in the vehicle group of 4T1 tumors, MVD was marginally reduced with Y332D and further decreased progressively following RT and the combination of RT with Y332D (Figure 5g).

### 2.6 Y332D Further Promoted the Formation of an Inflamed “hot” TME Remodeled by RT

**Figure 6:**
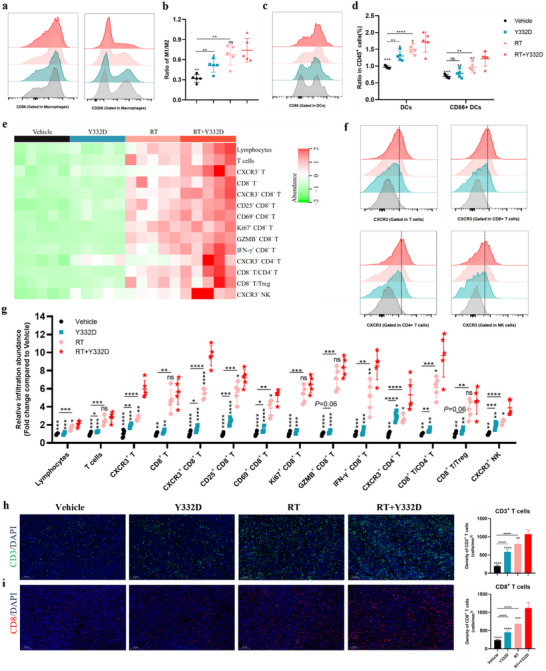
RT combined with Y332D enhanced intratumoral abundances of immune cells in the 4T1 tumor model. a,b) Flow cytometry assays assessed the expression of CD86 and CD206 in macrophages across treatment groups, as well as the ratio of M1 to M2 macrophages (*n =* 5). c,d) Flow cytometry assays evaluated the CD86 expression in DCs across treatment groups and the percentage of DCs and CD86+ DCs in CD45+ cells (*n =* 5). e) Heatmap depicting the relative abundance of tumor‐infiltrating lymphocytes (TILs) by flow cytometry assays (*n =* 5). f) Flow cytometry assays showing the expression of CXCR3 in T cells, CD4+ T cells, CD8+ T cells, and NK cells across treatment groups. g) Dot plot showing the relative abundances of TILs within the TME as assessed by flow cytometry assays (*n =* 5). h, i) Representative IF images and quantification of CD3 and CD8 staining are presented (*n =* 5). The scale bar in the IF images is 100 µm. All data are mean±SD. Statistical analyses were conducted using Student's *t*‐test. Statistical significance compared to the RT combined with the Y332D group is denoted as follows: *p < 0.05, **p < 0.01, ***p < 0.001, and ****p < 0.0001; ns: not significant.

To evaluate the impact of RT synergizes with Y332D on the tumor immune microenvironment (TIME) in the 4T1 tumor model with poor immune cell infiltration, we first examined the intratumoral infiltration of immune cells through spectral flow cytometry (Figure S4a,b, Supporting Information). Although the percentage of macrophages (CD11b+ F4/80+) and M1‐type macrophages (CD86+ CD11b+ F4/80+) in the RT plus Y332D therapy was not significantly higher compared to the monotherapies and control groups (Figure S4c, Supporting Information), the percentage of M2‐type macrophages (CD206+ CD11b+ F4/80+) was markedly reduced, leading to a significantly elevated M1/M2 ratio in RT plus Y332D therapy (**Figures** **6**a,b and S4c, Supporting Information). Meanwhile, RT plus Y332D significantly increased the density of DCs and CD86+ DCs versus either monotherapy or vehicle (Figure 6c,d). Compared to the vehicle and Y332D monotherapy, the RT plus Y332D therapy significantly increased the tumor‐infiltrating abundance of lymphocytes, CD3+ T, CD8+ T, activated CD8+ T (CD25+/CD69+ CD8+), cytotoxic CD8+ T (Gzmb+ CD8+ T and IFN‐γ+ CD8+ T), and proliferating CD8+ T (Ki67+ CD8+ T) cells (Figure 6e,g). Besides, the CD8+ T/CD4+ T ratio was significantly higher in the RT plus Y332D therapy versus Y332D or RT monotherapies (Figure 6e,g). Similarly, the CD8+ T/Treg ratio was significantly higher in the RT plus Y332D therapy versus vehicle or Y332D monotherapy (Figure 6e,g). Moreover, RT plus Y332D enhanced the abundance of activated NK (CD69+ NK) cells versus vehicle or monotherapies (Figure S4d, Supporting Information). Notably, RT plus Y332D markedly enhanced the infiltration of CXCR3+ T, CXCR3+ CD8+ T, CXCR3+ CD4+ T, and CXCR3+ NK cells versus either monotherapies or vehicle (Figure 6e–g), potentially due to the recruitment of CXCR3‐positive lymphocytes into the tumor by CXCL10 secreted by tumor cells, which is promoted by RT. In parallel, IF assays showed that RT plus Y332D significantly increased the density of intratumoral CD3+ T and CD8+ T cells versus either monotherapies or vehicles (Figure 6h,i).

### 2.7 RT Combined with Y332D Therapy Induced a Tumor Gene Expression Alteration that is Differentiated from that of Monotherapies

**Figure 7:**
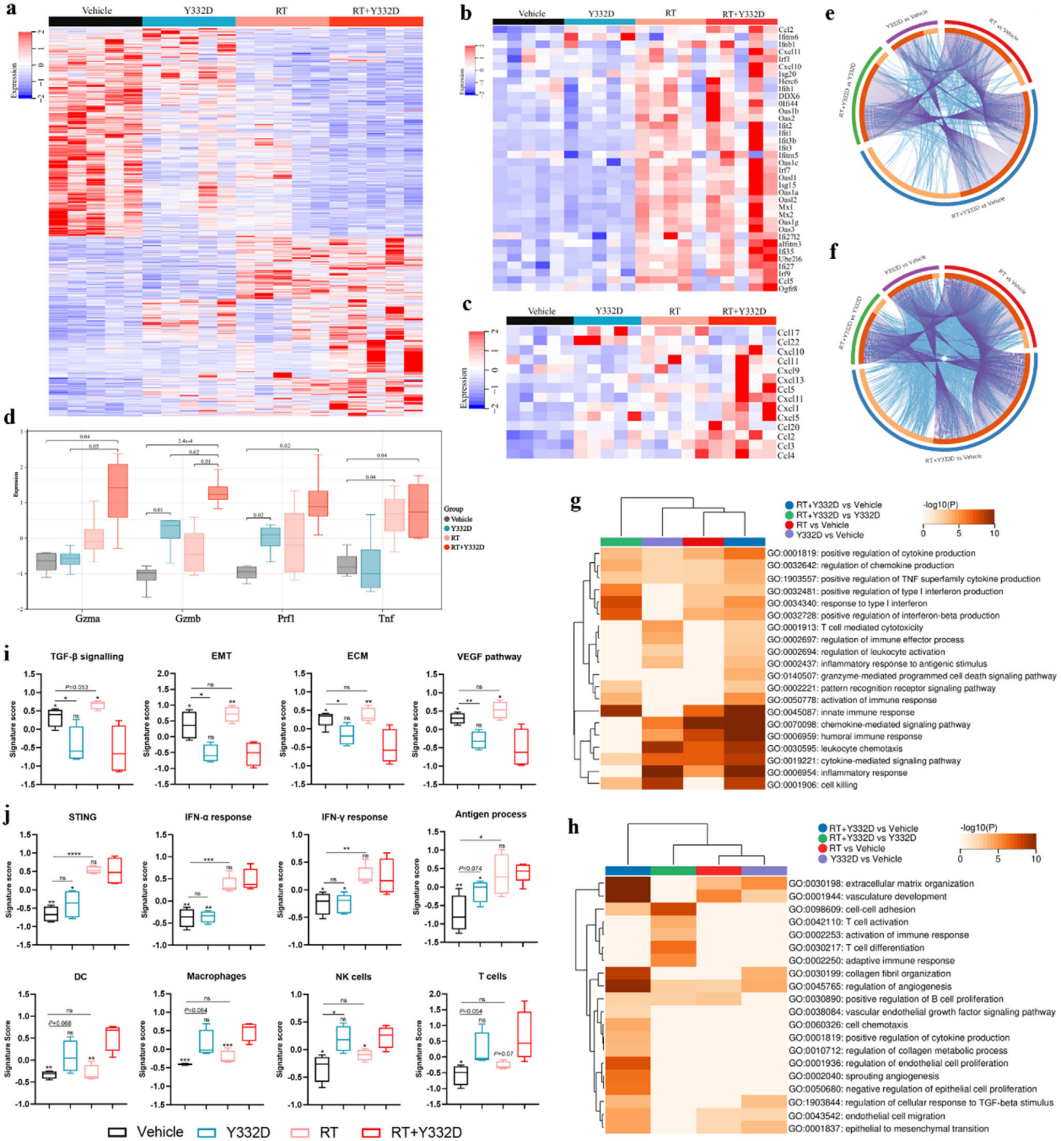
RT combined with Y332D induced a tumor gene expression alteration that was differentiated from that of monotherapies as revealed by RNA‐seq. a) Heatmap depicting the relative expression of differentially expressed genes (DEGs) among various treatment groups (*n =* 5). b, c) Heatmap illustrating the relative expression of ISGs and proinflammatory chemokines among treatment groups (*n =* 5). d) Boxplot presenting the expression levels of cytotoxicity‐related genes (Gzma, Gzmb, Prf1, Tnf) across treatment groups (*n =* 5). e, f) Circos plot demonstrating the overlap at the gene and pathway level between up‐regulated or down‐regulated DEGs from pairwise comparisons between treatment groups, respectively. Purple curves link shared genes and blue curves link genes that belong to the same enriched ontology term. The inner circle represents gene lists, genes that hit multiple lists are colored in dark orange, and genes unique to a list are shown in light orange. g, h) Heatmap of the GO enriched terms of up‐regulated or down‐regulated DEGs from pairwise comparisons between treatment groups, respectively. Colored by p‐values. i, j) Quantification of gene expression signatures associated with TGF‐β signaling, EMT, ECM, VEGF pathway, STING, IFN‐α response, IFN‐γ response, antigen processing, DCs, macrophages, NK cells, and T cells (*n =* 5). Signature scores are presented as boxplots. Statistical analyses were conducted using Student's *t*‐test. Statistical significance compared to the RT combined with Y332D group is indicated as follows: *p < 0.05, **p < 0.01, ***p < 0.001, and ****p < 0.0001; ns: not significant.

To elucidate the gene alterations induced by RT plus Y332D treatment, we performed RNA‐seq on 4T1 tumor tissues after different treatments. Pairwise comparisons among the treatment groups revealed that RT contributed to most, but not all, of the gene expression changes observed in the RT plus Y332D therapy group (**Figure** **7**a). Notably, RT combined with Y332D therapy mitigated expression levels of a subset of genes upregulated by RT, suggesting a potential reversal of RT‐induced gene expression by Y332D (Figure 7a). Moreover, compared to the vehicle, RT plus Y332D therapy resulted in a significant upregulation of ISGs, pro‐inflammatory chemokines (Ccl2, Ccl3, Ccl4, Ccl5, Ccl11, Ccl20, Cxcl1, Cxcl5, Cxcl9, Cxcl10, Cxcl11, and Cxcl13), and cytotoxicity‐related genes (Gzma, Gzmb, Prf1, and Tnf) (Figure 7b–d). To gain a comprehensive understanding of why the RT plus Y332D treatment is more effective than monotherapies at the gene transcription level, we conducted gene ontology (GO) biological process (BP) pathway enrichment analysis for both up‐regulated and down‐regulated Differentially expressed genes (DEGs) in pairwise comparisons between the treatment groups, respectively. The up‐regulated DEGs were mainly enriched in multiple immune‐related pathways, such as inflammatory response, innate immune response, humoral immune response, activation of the immune response, regulation of cytokine and chemokine production, cytokine/chemokine‐mediated signaling pathway, cellular response to IFN‐β, immune response‐activating signal transduction, regulation of type I interferon production and response to type I interferon, leukocyte chemotaxis and activation, regulation of immune effector process, T cell‐mediated cytotoxicity, pattern recognition receptor signaling pathway, cell killing, etc (Figure 7e,g). Especially, these pathways were most significantly enriched in the RT combined with Y332D therapy (Figure 7g). Conversely, the down‐regulated DEGs were predominantly enriched in tumor stroma remodeling‐related pathways, such as extracellular matrix organization, collagen fibril organization, regulation of collagen metabolic process, regulation of cellular response to TGF‐beta stimulus, EMT, vasculature development, and regulation of angiogenesis, endothelial cell migration, regulation of endothelial cell proliferation, sprouting angiogenesis, VEGF signaling pathway, negative regulation of epithelial proliferation, etc (Figure 7f,h). Similarly, most of these pathways were most significantly enriched in the RT combined with Y332D therapy (Figure 7h).

To more intuitively evaluate the impact of RT plus Y332D therapy on the TME, we quantified the expression of gene signatures involved in tumor stromal remodeling and immune activation panels. In the tumor stromal remodeling panel (Figure 7i), Y332D significantly decreased the scores of TGF‐β signaling, EMT, ECM, and VEGF pathway signatures compared to the vehicle, while RT showed a trend toward increasing these scores versus the vehicle. The RT plus Y332D therapy decreased the scores of all these signatures relative to RT (Figure 7i). In the immune activation panel (Figure 7j), RT strongly upregulated the expression scores of STING‐, IFN‐α‐, IFN‐γ‐, and antigen process‐associated signatures. Meanwhile, RT plus Y332D significantly increased these signature scores versus vehicle and Y332D, but not versus RT. In this panel (Figure 7j), Y332D significantly increased the scores of NK cells signature and slightly increased the scores of DCs, macrophages, and T cells signature versus vehicle. Although RT had no significant effect on these four signatures, RT plus Y332D significantly increased these signature scores versus vehicle and RT, but not versus Y332D, which might be attributed to the early time point of sampling (3 days after RT). At the time of sampling, it is plausible that the RT regimen for three consecutive days had effectively ablated the resident immune cells within the TME, while the anticipated influx of immune cells as a consequence of RT had not yet appeared.

### 2.8 RT Combined with Y332D Therapy Potentiated the Abscopal Effect on Spontaneous 4T1 Lung Metastatic Disease Via Increasing Lymphocytes and Decreasing MDSCs in Metastatic Lungs

**Figure 8:**
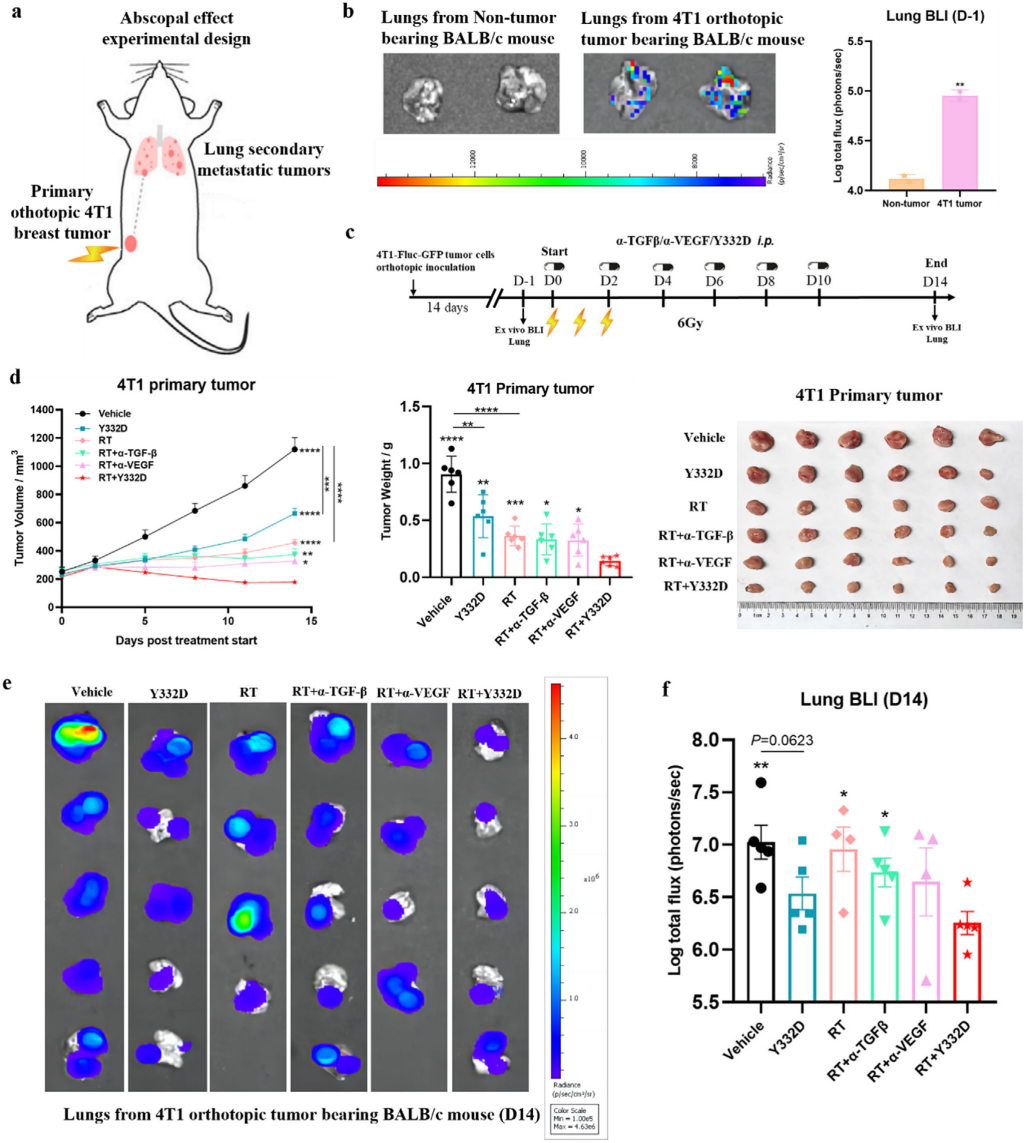
RT plus Y332D therapy induces abscopal regression of spontaneous 4T1 lung metastases. a) Schematic representation of the experimental design. b) Ex vivo bioluminescence imaging (BLI) of lung metastases at day ‐1, with corresponding lung images and quantification data (*n =* 2). c) Schematic diagram indicating the schedule and time points for the evaluation of secondary metastases and the administration of treatments. d) Tumor growth curves, tumor weights, and photographs of orthotopic 4T1 primary tumors were displayed (*n =* 6). e, f) Lung images and quantification of Ex vivo BLI of lung metastases at days 14 for each treatment group (*n =* 4 or 5). All data are mean±SEM. Statistical analyses were conducted using Student's *t*‐test. Statistical significance compared to the RT combined with Y332D group is denoted as follows: *p < 0.05, **p < 0.01, ***p < 0.001, and ****p < 0.0001; ns: not significant.

Although the abscopal effect induced by RT alone is infrequent in clinical practices, the combination of RT with immunomodulators like Y332D provides an opportunity to augment the abscopal response and extend the use of RT for the treatment of both local and metastatic disease.^[^ ^21^ ^]^ Utilizing luciferase‐expressing 4T1 cells and Bioluminescence Imaging (BLI), we investigated whether the combination of RT and Y332D could enhance antitumor immunity and ameliorate established spontaneous lung metastatic diseases that are not directly targeted by RT (**Figure** **8**a). The establishment of a secondary tumor was confirmed in the lungs of tumor‐bearing mice 14 days after tumor inoculation (day ‐1) using ex vivo BLI, while no luminescence was detected in healthy lungs (Figure 8b). On the following day (day 0), the mice were systemically treated with Y332D, α‐TGF‐β, and α‐VEGF, as well as the primary tumor received local RT (Figure 8a,c). Both Y332D and RT monotherapies significantly inhibited primary orthotopic tumor growth, and the RT plus Y332D therapy further suppressed primary tumor growth compared to either monotherapy (Figure 8d), suggesting a synergistic effect in controlling primary tumor growth. Moreover, the combination of RT and Y332D was significantly more effective than RT with α‐VEGF or α‐TGF‐β in inhibiting primary tumor growth and reducing tumor burden (Figure 8d). Compared to the vehicle, Y332D slightly reduced lung BLI on day 14, whereas RT had no significant effect (Figure 8e,f). However, RT plus Y332D therapy significantly reduced lung BLI on day 14 versus vehicle, RT, or RT plus α‐TGF‐β, but not versus Y332D monotherapy (Figure 8e,f). These findings indicated that the systemic effect of Y332D reduces lung metastases and facilitates abscopal effects of RT against lung metastatic disease in RT plus Y332D therapy.

**Figure 9:**
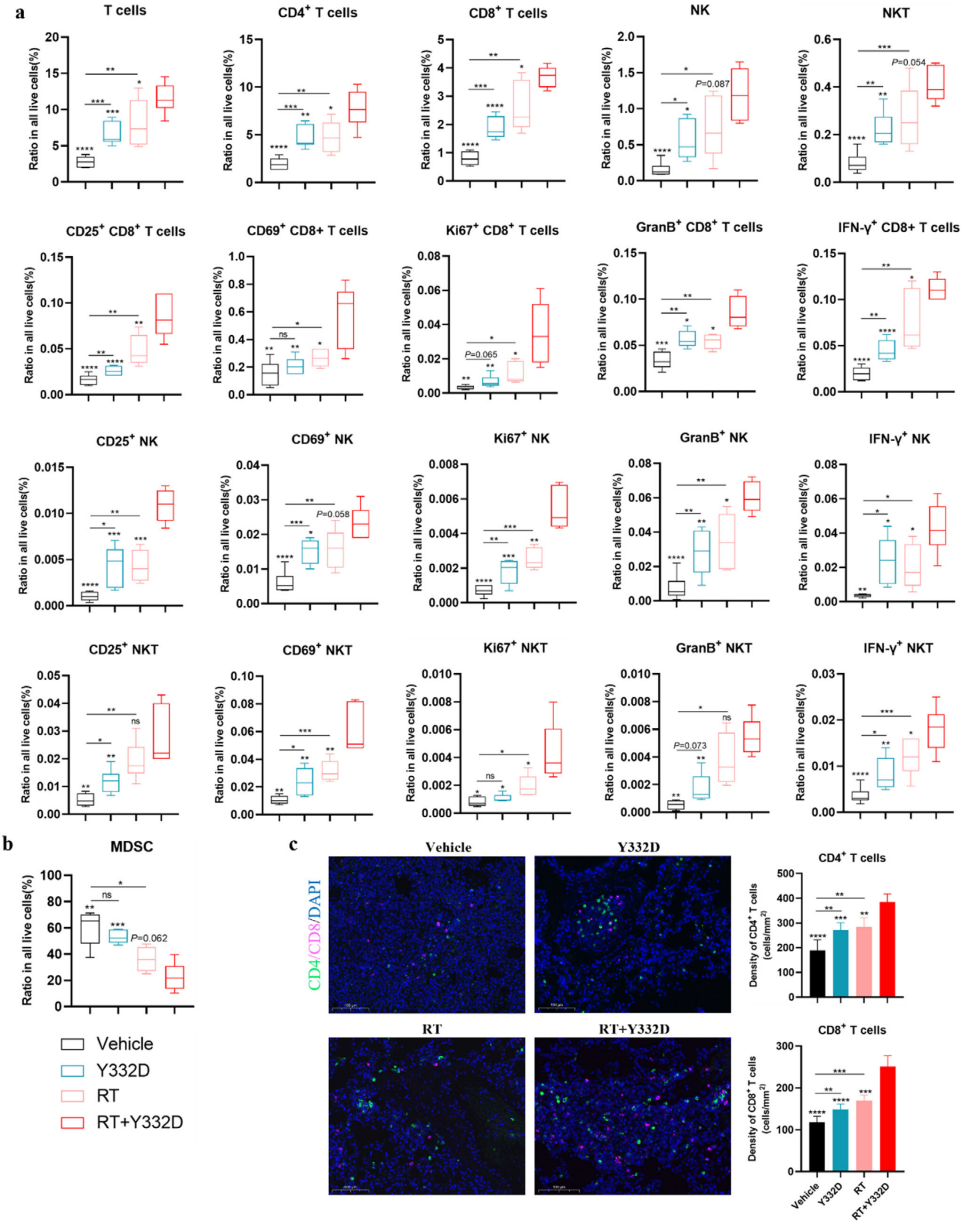
The “in‐situ vaccines” in primary tumors induced by RT plus Y332D therapy enhanced lymphocytes infiltration in lung metastases. a,b) Quantification of various immune cell populations infiltrating the lungs, as assessed by flow cytometry assays, including CD3+ T cells, CD4+ T cells, CD8+T cell, CD69+CD8+T cell, CD25+CD8+T cell, Ki67+CD8+T cell, Granzyme‐B+CD8+T cell, IFN‐γ+CD8+T cell, NK cell, CD69+NK cell, CD25+NK cell, Ki67+NK cell, Granzyme‐B+NK cell, IFN‐γ+NK cell, NKT cell, CD69+NKT cell, CD25+NKT cell, Ki67+NKT cell, Granzyme‐B+NKT cell, IFN‐γ+NKT cell, and MDSCs. The proportion of infiltrating immune cells in the total live cells was calculated (*n =* 5). Data are presented as boxplots. c) Representative IF images and quantification of infiltrating CD4+ and CD8+ T cells in metastatic lung lesions (*n =* 5). Data are mean±SD. The scale bar in the IF images refers to 100 µm. Statistical analyses were conducted using Student's *t*‐test. Statistical significance compared to the RT combined with Y332D group is indicated as follows: *p < 0.05, **p < 0.01, ***p < 0.001, and ****p < 0.0001; ns: not significant.

The abscopal effects induced by RT are likely mediated through the induction of antitumor immunity, which is driven, in part, by RT‐induced tumor inflammation and antigen release.^[^ ^53^ ^]^ To elucidate the mechanisms underlying the abscopal effects induced by RT plus Y332D therapy, we analyzed the single‐cell suspensions of whole lung tissues from mice bearing 4T1 orthotopic tumors using flow cytometry. Relative to the vehicle and monotherapies, the combination of RT and Y332D therapy increased the percentage of infiltrating CD3+ T, CD4+ T, CD8+ T, activated CD8+ T (CD25+ CD8+ and CD69+ CD8+), cytotoxic CD8+ T (Gzmb+ CD8+ T and IFN‐γ+ CD8+ T), proliferating CD8+ T (Ki67+ CD8+ T), NK, activated NK (CD25+ NK and CD69+ NK), cytotoxic NK (IFN‐γ+ NK and Gzmb+ NK), proliferating NK (Ki67+ NK), NKT, activated NKT (CD25+ NKT and CD69+ NKT), cytotoxic NKT (IFN‐γ+ NK and Gzmb+ NK), and proliferating NKT (Ki67+ NKT) in total lung tissues (**Figure** **9**a). Consistently, lung IF showed that the density of CD8+ T cells and CD4+ T cells in metastatic lesions significantly increased with Y332D versus control, and further increased with RT plus Y332D therapy versus Y332D or RT (Figure 9c). Previous studies have reported that pulmonary myeloid cell infiltration promotes 4T1 lung metastatic disease and that pulmonary MDSC infiltration increases during 4T1 disease progression.^[^ ^54^, ^55^ ^]^ Flow cytometry analysis revealed RT plus Y332D therapy significantly decreased the percentage of MDSCs in the lung (Figure 9b).

## 3 Discussion

Immunotherapies, particularly ICIs, have revolutionized the treatment landscape for various cancers over the last few decades. Nevertheless, the majority of patients do not respond to these therapies. The varying immunotherapy response rate can be in part explained by the intra‐ and inter‐tumor heterogeneity resulting in a high diversity of immune escape mechanisms induced by the tumor.^[^ ^56^, ^57^ ^]^ Based on T‐cell infiltration, the TME has been categorized into three immune phenotypes that strongly correlate to the response to immunotherapy. Among these, the immune‐inflamed phenotype, or “hot” tumor, characterized by robust T cell infiltration, is more often responsive to immunotherapies. In contrast, the immune‐desert and immune‐excluded phenotypes, also known as “cold” tumors, are non‐inflamed tumors that typically fail immunotherapies. The immune escape mechanisms in non‐inflamed tumors usually include poor tumor immunogenicity, APCs dysfunction, and hampered T cell priming and activation.^[^ ^58^ ^]^ Also, hypoxic environments and interstitial‐dependent exclusion contribute to immune escape in non‐inflamed tumors.^[^ ^59^ ^]^

RT is known to have profound immunomodulatory effects and represents a promising modality to reprogram the TME. As such, it is increasingly viewed as a valuable combination partner with ICIs and other immuno‐oncology agents.^[^ ^60^ ^]^ Primarily, RT‐induced tumor cell micronuclei activate the cytosolic nucleic acid sensor cGAS‐STING pathway in tumors, which induces type I interferon (IFN) production, and propagation of the resulting inflammatory signals remodels the immune contexture of the TME.^[^ ^13^ ^]^ However, at doses between 12 and 18 Gy, RT induced the cytoplasmic deoxyribonuclease Trex1, which degrades cytoplasmic double‐stranded DNA, therefore preventing the activation of the GAS‐STING pathway.^[^ ^61^ ^]^ In our study, we found that a moderate dose of 6 Gy irradiation activated the cGAS‐STING pathway and triggered type I IFN response both in vitro and in vivo, leading us to adopt a medium dose of 6 Gy as the single irradiation dose in subsequent studies. Meanwhile, increased expressions of IFN‐inducible chemokines CCL2, CCL5, and CXCL10 at both mRNA and protein levels were observed following RT, contributing to the recruitment of monocytes, NK cells, and effector T cells to the irradiated tumor.^[^ ^62^, ^63^ ^]^ Besides, under radiation, most tumor cells undergo apoptosis, necrosis, autophagy, or mitotic catastrophe, during which neoantigens are expressed, and tumor antigens are effectively exposed.^[^ ^9^ ^]^ In parallel, RT induces ICD and stressed and dying cells to release cytoplasmic ATP and HMGB1 within minutes.^[^ ^64^ ^]^ Extracellular ATP, binding to the purinergic receptor P2X7, acts as a prominent “find‐me” signal for DC precursors, facilitating the recruitment of myeloid monocytes to sites of active ICD.^[^ ^65^ ^]^ Tumor cell's surface‐exposed CRT serves as an “eat‐me” signal that facilitates the phagocytosis of dying cells or their corpses by DCs or their precursors, leading to the maturation of DCs and subsequently activates the production of pro‐inflammatory cytokines like IL‐6 and TNF‐α in APCs.^[^ ^66^, ^67^ ^]^ Extracellular HMGB1 released from tumor cells binds to TLR4 and TLR9 on DCs, promoting efficient processing and cross‐presentation of tumor antigens and stimulating DCs maturation.^[^ ^68^, ^69^ ^]^ Our study also demonstrated that RT‐induced ICD in multiple tumor cells in vitro. Moreover, RT can up‐regulate MHC‐I molecules on the surface of tumor cells, resulting in increased cytotoxic T lymphocyte recognition of irradiated cells,^[^ ^17^, ^70^ ^]^ which was confirmed by our results. In a word, our findings confirmed that RT activated the cGAS‐STING pathway, triggered type I IFN response, induced ICD in tumor cells, and upregulated tumor cell surface MHC‐I, thus priming anti‐tumor immunity and restoring immune recognition.

However, the immunostimulatory effects of RT may be counterbalanced by immunosuppressive mechanisms caused by RT, such as RT‐induced TGF‐β and VEGF. Our findings, consistent with previous studies, revealed that both TGF‐β and VEGF levels were elevated not only in irradiated tumor cells and tissues but also in the plasmas of mouse‐bearing irradiated tumors. RT‐induced TGF‐β and VEGF expression can impede intratumoral immune cells infiltration by enhancing peritumoral collagen deposition and tumor angiogenesis, thereby counteracting the immunostimulatory effects of RT. Therefore, the simultaneous blockade of TGF‐β and VEGF with Y332D can ameliorate these effects by reducing abnormal collagen accumulation and vascular barriers and can reverse tumor‐intrinsic and RT‐induced TGF‐β‐ and VEGF‐dependent ECM remodeling, EMT, neovascularization, radioresistance, and immunosuppression. Moreover, our in vitro experiments also confirmed that simultaneously blocking TGF‐β and VEGF with Y332D sensitized tumor cells to RT. Consequently, we formulated a combination strategy to resensitize tumors with poorly immunogenic, stroma‐rich, and active angiogenesis through multiple complementary mechanisms of RT and Y332D. Our efficacy studies demonstrated that RT combined with Y332D therapy exhibited potent antitumor activity in multiple murine tumor models, outperforming Y332D or RT monotherapies and RT combined with α‐TGF‐β or α‐VEGF therapy. IF and RNA‐seq data indicated that Y332D reversed the uptrend of RT‐induced TGF‐β signaling, ECM deposition, EMT process, and VEGF pathway within the combination treatment group. Additionally, Y332D effectively inhibited spontaneous lung metastasis in 4T1 orthotopic breast tumors. The explorations of the TIME in the 4T1 tumor model revealed that RT combined with Y332D therapy significantly promoted DCs maturation and macrophage polarization toward an M1‐like phenotype, as well as enhanced the abundance, activation, proliferation, and cytotoxicity of CD8+ T cells and the activation of NK cells within the TME. In the antitumor immune cycle triggered by RT, DCs are responsible for the uptake of tumor antigens and the activation of CD8+T cells, which migrate and infiltrate tumor tissues to specifically recognize and kill tumor cells, thereby initiating the adaptive immune response; NK cells rapidly initiate the innate immune response without antigen presentation; M1‐type macrophages coordinately attack tumors through secreting proinflammatory cytokines.^[^ ^20^, ^58^, ^64^ ^]^

**Figure 10:**
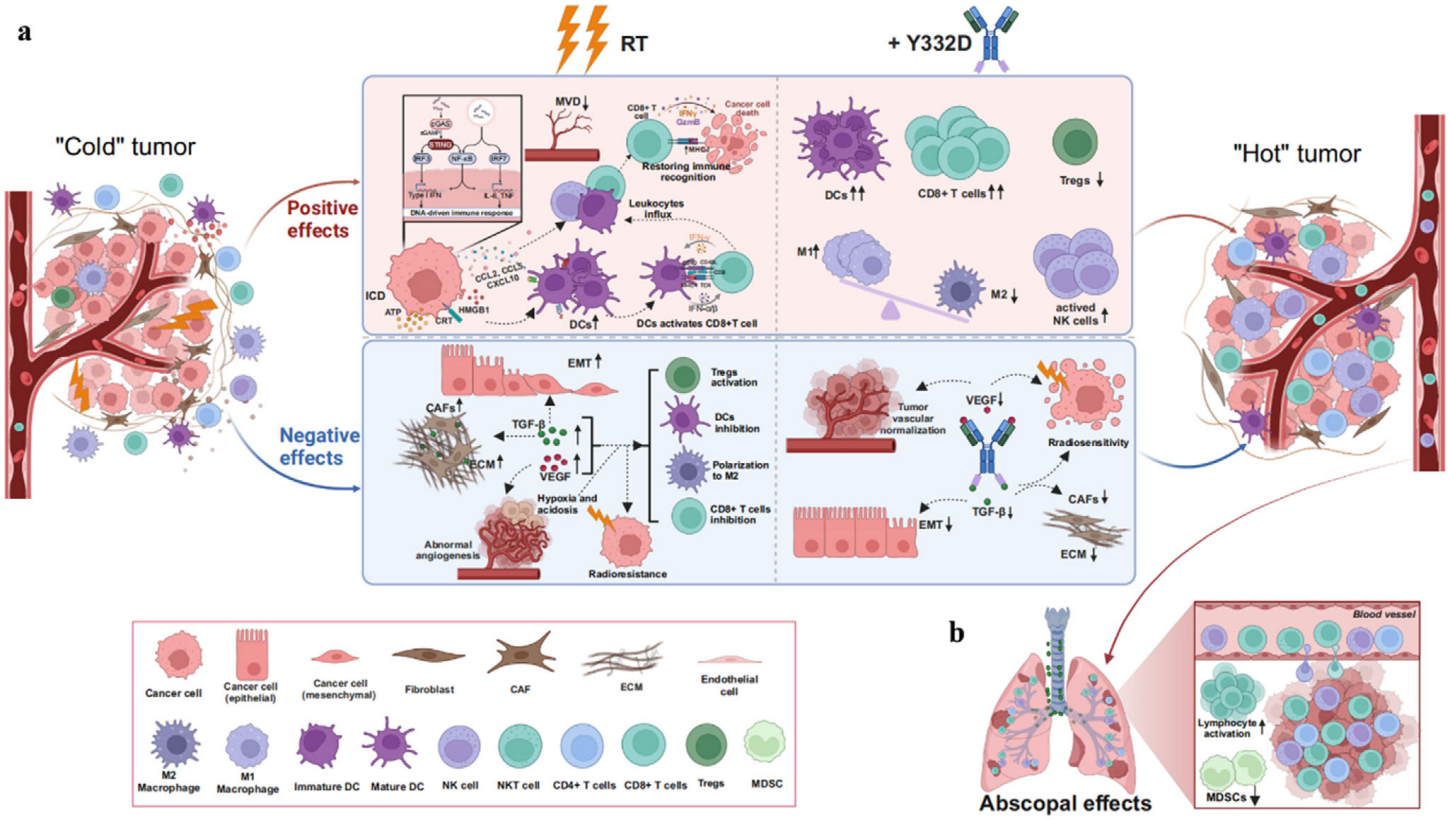
Schematic illustrating the synergistic mechanism of RT combined with Y332D in the TME and abscopal metastatic niches. a) The positive effects of RT (depicted above in a light orange area) are further enhanced or complemented by Y332D. Moreover, Y332D counterbalanced the negative effects of RT (illustrated below in a light blue area), which are associated with the upregulation of TGF‐β‐ and VEGF‐mediated epithelial‐mesenchymal transition (EMT), extracellular matrix (ECM) remodeling, aberrant angiogenesis, immunosuppression, and radioresistance. b) The TME reprogramming by Y332D and local RT forms an effective in situ tumor vaccine that amplifies the antitumor immune response, leading to regression of preestablished, spontaneous lung metastatic lesions. This abscopal effect was attributed to an enhanced lymphocyte influx and a reduction of immunosuppressive MDSCs in metastatic lungs.

Heretofore, we have demonstrated the immunostimulatory effects of RT acting on the primary irradiated tumors, and Y332D potentiated these effects and reversed negative RT effects by blocking the effects of TGF‐β and VEGF (**Figure** **10**a). It is widely accepted that in‐situ vaccines induced by RT are the key mechanism for transforming local effects into abscopal responses. The activated CD8+ T cells and NK cells in primary irradiated tumors migrate to metastases via blood circulation, thereby controlling secondary tumor growth at no‐irradiated sites.^[^ ^71^ ^]^ However, the abscopal effect induced by RT alone is rare in preclinical and clinical practices, which may be caused by RT‐induced immunosuppressive mediators in primary tumors, such as TGF‐β and VEGF, into the circulation to exert systemic immunosuppressive effects. Our study demonstrated increased levels of TGF‐β and VEGF in the plasma of mice bearing irradiated cancers, suggesting that Y332D could amplify abscopal effects induced by RT via blocking the systemic effects of TGF‐β and VEGF. Indeed, our findings showed that RT plus Y332D therapy significantly inhibited lung metastatic tumor growth compared to vehicle or RT. Lung flow cytometry and IF revealed that RT plus Y332D therapy enhanced lymphocytes (CD3+ T cells, CD4+ T cells, CD8+ T cells, NK cells, and NKT cells) in the metastatic lung. Moreover, RT combined with Y332D significantly promoted the activation, proliferation, and cytotoxicity of CD8+ T cells, NK, and NKT cells and decreased the MDSCs in metastatic lungs. Prior research has indicated that pulmonary MDSCs infiltration supports metastatic growth by promoting tumor cell proliferation.^[^ ^54^ ^]^ Another study has shown that MDSCs alter the premetastatic lung into an inflammatory and proliferative environment, diminish immune protection, and promote metastasis through aberrant vasculature formation.^[^ ^55^ ^]^ Our results therefore suggested that the abscopal response induced by RT combined with Y332D is mediated, in part, through the reduction of tumor proliferation‐ and metastasis‐promoting MDSCs. Targeting MDSCs has been shown to significantly improve treatment outcomes and ameliorate lung metastatic lesions.^[^ ^72^ ^]^ Our results indicated that this combination strategy successfully overcame the negative effects caused by RT and effectively amplified the positive immune priming effects of RT, leading to the regression of distant metastatic tumors (Figure 10b).

Leveraging its unique ability to simultaneously block two critical signaling pathways in the TME, Y332D shows broad application potential in solid tumors characterized by abundant tumor stroma and active angiogenesis. The core mechanism of Y332D lies in its synergistic action of tumor vascular normalization, immune remodeling, and tumor stromal regulation, thereby achieving a durable antitumor effect. This preclinical work is a fundamental study in exploring potential combination strategies of Y332D in the clinic. However, Y332D may have the following limitations or drawbacks: First, its large molecular weight (≈170 kDa) makes it difficult to penetrate dense tumor stroma, resulting in insufficient intratumoral drug concentrations. Second, Y332D does not target tumor‐specific antigens, which may lead to accumulation in normal non‐target tissues and increased toxicity. Finally, long‐term use may lead to adaptive resistance. Therefore, the actual efficacy and safety of Y332D remain to be determined by future clinical studies.

## 4 Conclusion

In this study, we demonstrated a synergistic antitumor activity of RT plus Y332D, where positive effects of RT, such as the activation of the cGAS‐STING pathway, induction of IFN type I response, and ICD, augmented influx of leukocytes, enhanced antigen presentation, increased T cell activation, and antiangiogenic effects, are further enhanced or complemented by Y332D. Moreover, Y332D counteracted negative RT effects, leading to local TME reprogramming and enhanced immune responses. TME remodeling by Y332D with local tumor RT forms an effective in‐situ tumor vaccine that amplifies antitumor immune response for effective local tumor eradication and an enhanced abscopal effect against preestablished, spontaneous lung metastatic lesions. This abscopal effect was attributed to the enhanced influx and activation of CD8+ T cells, NK cells, and NKT cells, as well as the reduction of immunosuppressive MDSCs within metastatic lungs.

## 5 Experimental Section

### Cell lines and Therapeutic Antibodies

Murine mammary carcinoma 4T1, colon carcinoma CT26, and hepatoma H22 cell lines, derived from BALB/c mice, were cultured in RPMI‐1640 (Gibco) medium supplemented with 10% fetal bovine serum (FBS) (Gibco). The glioma 261 (GL261) cell line, derived from C57BL/6 mice, was cultured with DMEM (Gibco) containing 10% FBS. The therapeutic antibodies, including α‐VEGF, α‐TGF‐β, and the bsAb Y332D (targeting VEGF/TGF‐β), utilized in this study were generously provided by Wuhan YZY Biopharma Co., Ltd.

### Phospho‐Specific Flow Cytometry (phosflow)

For the detection of intracellular phosphoprotein in tumor cells, the phosflow assay was employed. Irradiated or non‐irradiated tumor cells were detached using trypsinization, followed by fixed with prewarmed phosflow fix buffer I (557870, BD Biosciences) at 37 °C for 10 min, centrifuged, and the supernatant was removed. The cells were then permeabilized with ice‐cold Phosflow perm/wash buffer I (557885, BD Biosciences) for 20 min on ice, washed twice, centrifuged, and the supernatant was removed. Cells were resuspended in the staining buffer for intracellular phosphoprotein staining. Anti‐mouse phospho‐TBK1 Rabbit mAb (5483, CST, 1:500), phospho‐IRF‐3 Rabbit mAb (29047, CST, 1:500), or γ‐H2AX Rabbit mAb (9718, CST, 1:400) was added to each tube and incubated at room temperature for 1 h. After centrifugation in 1X PBS to wash and discard the supernatant, cells were resuspended in diluted fluorochrome‐conjugated secondary antibody (405307, BioLegend, 1:500) and incubated for 30 min at room temperature in the dark. Samples were finally resuspended in 100µl staining buffer and analyzed using a BD Accuri C6 flow cytometer. FACS data were processed with FlowJo_V10 software.

### Real‐time Quantitative PCR (RT‐qPCR)

Total RNA was extracted using the Animal Tissue/Cell Total RNA Extraction Kit (DP451, TIANGEN) following the manufacturer's instructions and quantified using a Nanodrop UV–Vis Spectrophotometer (Thermofisher). The synthesis of RNA to complementary DNA (cDNA) was conducted employing dNTPs and M‐MLV RT reverse transcriptase (M1701, Promega) according to the manufacturer's protocol, and the resulting cDNA was diluted to a standardized concentration for mRNA expression analysis. RT‐qPCR was conducted with the iQ SYBR Green Supermix (1708882, Bio‐Rad) and pre‐designed primers (Tsingke Biotech) on the Applied Biosystems 7500 PCR System (Applied Biosystems, USA) according to the manufacturer's protocol. Before experiments, primer efficiency tests and melt curve analyses were performed to ensure the specificity of amplified products from all primer pairs. GAPDH was employed as a housekeeping gene to normalize the cycle threshold (Ct) values for the individual genes analyzed. The relative expression levels of each gene were determined using the 2^−ΔΔCT^ [ΔΔ*Ct* = Δ*Ct* (test)−Δ*Ct* (control)] method. The primer sequences utilized in this study are detailed in Table S1 (Supporting Information).

### The Realtime‐Glo Extracellular ATP Assay

The RealTime‐Glo Extracellular ATP Assay (M1701, Promega) was a bioluminescent assay designed for kinetic monitoring of ATP released from dying, stressed, or activated cells. The ATP released from irradiated or non‐irradiated tumor cells reacted with the UlGlo luciferase in the reagents to generate a luminescent signal, thereby assessing ATP release.

### Cytokines and Chemokines Detection

The concentrations of murine CXCL10 and HMGB1 in supernatants of irradiated or non‐irradiated cells were quantified using the Mouse CXCL10/IP‐10 ELISA Kit (abs520013, Absin) and the Mouse HMGB1 ELISA Kit (E‐EL‐M0676, Elabscience), respectively. Additionally, the levels of mouse TGF‐β1 and VEGF in cell supernatants, tumor tissue homogenates, and plasma were assessed using the Mouse TGF‐β1 Precoated ELISA kit (DKW12‐2710‐096, Dakewe) and the Mouse VEGF Quantikine ELISA Kit (MMV00, R&D), respectively. The level of IFN‐β in cell supernatants and tumor tissue homogenates was measured using the Mouse IFN‐β Quantikine ELISA Kit (MIFNB0, R&D). The levels of murine CCL2 and CCL5 in cell supernatants were determined using multiplex fluorescence–encoded beads (740451, BioLegend). Cytokines detection in the co‐culture supernatants was performed using Cytometric Bead Array (CBA) (560485, BD). All procedures were executed following the manufacturers' guidelines.

### Co‐Culture Assay

To simulate the RT‐induced ICD and its associated tumor‐specific cytotoxic effects in vitro, 4T1‐Fluc cells (target cells), irradiated or non‐irradiated 4T1 cells, and splenocytes cells from BALB/c mice (effector cells) were co‐cultured. The experimental procedure was illustrated in Figure S1b (Supporting Information). On day 1, 4T1‐FLuc and 4T1 parental tumor cells were seeded into 24‐well plates and T25 flasks, respectively, overnight. On day 2, 4T1 parental cells were irradiated with 6Gy or non‐irradiated. RT induced ICD in the parental 4T1 tumor cells, enhancing cross‐presentation and co‐stimulation by dendritic cells to CD8+ T cells. 4 h later, both irradiated or non‐irradiated 4T1 parental cells, along with their respective culture media, were collected and added to the adherent 4T1‐FLuc cell plate at a ratio of 1:1. Meanwhile, the spleen from BALB/c mice was harvested to create single cell suspension, which was added to the 4T1‐FLuc cell culture plate at a 1:5 ratio. Subsequently, 10^6^ pm antibodies were added to the co‐culture system. After 3 days of co‐culture, the supernatants were collected for cytokines detection, dead cells were washed off, and 4T1‐Fluc cells viability was assessed using a luciferase assay (Bio‐Glo Luciferase, G7941, Promega) according to the manufacturer's recommendations.

### CCK‐8

Given the role of TGF‐β and VEGF in promoting the radioresistance of tumor cells, CCK‐8 assays were performed to measure the radiosensitivity of tumor cells after Y332D antagonized the effect of TGF‐β and VEGF. The 4T1, CT26, H22, and GL261 cells (1 × 10^3^ per well) were seeded in 96‐well plates, followed by the addition of 10 ng mL^−1^ TGF‐β1, 10 ng mL^−1^ VEGFA, and 10^6^ pm antibodies. The next day, tumor cells were irradiated with 6 Gy. At 48 h post‐RT, cell viability was assessed using the CCK‐8 reagent (10 µL per well, LK04, Dojindo).

### γ‐H2AX IF Staining

The day before RT, 4T1 tumor cells (2 × 10^4^ per well) were seeded into 24‐well plates, followed by the addition of 10 ng mL^−1^ TGF‐β1, 10 ng mL^−1^ VEGFA, and 10^6^ pm antibodies. On the following day, 4T1 cells were irradiated with 6 Gy. At 4 h post‐RT, cells were fixed with 4% paraformaldehyde for 30 min, permeabilized with 1% Triton‐X 100 for 20 min, and incubated with 5% BSA for 1 h. Cells were then incubated with γ‐H2AX Rabbit mAb (9718, CST, 1:600) at 4°C overnight. Fluorescent secondary antibody (SA00003‐2, proteintech, 1:250) was incubated at room temperature for 1 h. Cell nuclei were counterstained with DAPI (C2035S‐6, Beyotime).

### Murine Tumor Models and Treatment

For efficacy studies, 1 × 10^6^ CT26 cells and 1 × 10^6^ H22 cells were subcutaneously injected into the right flank of BALB/c mice and 5 × 10^5^ 4T1 cells were orthotopically inoculated in the right second mammary fat pad of BALB/c mice. When the tumor volume (TV) reached ≈200 mm^3^, mice were randomized into six treatment groups (Vehicle, Y332D, RT, RT plus α‐TGF‐β, RT plus α‐VEGF, RT plus Y332D) according to TV (day 0) and treatment was initiated (*n =* 6 mice per group). TV was measured every other day or every two days and calculated using the formula: TV = (length  ×  width^2^)/2. Mice were euthanatized when TV exceeded 2000 mm^3^ or the study ended. For survival studies, 2 × 10^5^ GL261 cells or 1 × 10^5^ GL261 cells were intracranially inoculated in the right hemisphere of C57BL/6 mice. Mice were randomized into six groups (*n =* 9 or 11 mice per group). Mice were euthanized when they displayed signs of morbidity, including neurological symptoms due to tumor burden such as circling, seizure, lethargy, and ataxia. For abscopal effect studies, 4T1‐Fluc‐GFP (1 × 10^5^) cells were orthotopically inoculated into the right fourth mammary fat pad of BALB/c mice on day ‐14. On day ‐1, lungs from 4T1 tumor‐bearing mice and non‐tumor‐bearing mice were harvested and placed in D‐fluorescein (300 mg mL^−1^ saline) for ex vivo imaging to confirm the presence of tumor metastasis in the lungs. Then the mice were randomly divided into 6 groups according to the volume of the primary tumor and treatment was initiated (*n =* 6 mice per group). Mice were euthanized on day 14. For all studies, equivalent mole α‐VEGF (8.7 mg kg^−1^), α‐TGF‐β (6 mg kg^−1^), and Y332D (10 mg kg^−1^) were administrated on alternate days by intraperitoneal injection six times. Subcutaneous tumors and orthotopic 4T1 tumors were irradiated with three consecutive fractions of 6 Gy from day 0 to 2 and the mouse bearing‐orthotopic GL261 tumor received whole brain radiotherapy (WBRT) with a dose of 6 Gy on day 0 using the Small Animal Radiation Research Platform (SARRP, PXI X‐RAD 225Cx, CT, USA) with an X‐ray energy of 225 kV, a current beam of 13 mA, a dose rate of 1.3 Gy/min and a source‐surface distance (SSD) of 60 cm.

### Ex vivo BLI

In the orthotopic 4T1 model, lungs were harvested and immersed in D‐luciferin (ST196, Beyotime) for 2–3 min of imaging using an IVIS Spectrum (PerkinElmer) on days –1 and 14. BLI images were analyzed using Living Image 4.3.1 (PerkinElmer) software, and total flux (photons/sec) was calculated and exported for all regions of interest (ROIs).

### Flow Cytometry Assays

The expression of CRT and MHC‐I on the surface of tumor cells were detected by flow cytometry. Adherent cells were dissociated into single‐cell suspensions using 0.05% trypsin and 0.02% EDTA and stained with fluorochrome‐conjugated anti‐mouse calreticulin (62304, CST) and anti‐mouse MHC‐I (562003, BD) antibodies for 30 min at room temperature in the dark. After centrifugation in 1X PBS to wash and discard the supernatant, the cells were resuspended in 1X PBS and analyzed using a BD Accuri C6 or BD FACSCelesta flow cytometer. For tissues, 100mg of 4T1 tumors or lung tissues were weighed and then single‐cell suspensions were prepared through mechanical disruption and enzymatic digestion with collagenase B (0.5 mg mL^−1^, 11088807001 Roche), DNase I (0.05 mg mL^−1^, Sigma–Aldrich), and hyaluronidase (0.5 mg mL^−1^, H3506, Sigma‐Aldrich) at 37 °C for 1 h, followed by filtration through a 70‐µm nylon cell strainer. Cell suspensions were treated with red blood cell lysis buffer (C3702, Beyotime) and washed. Subsequently, the immune cells in the single‐cell suspension of 4T1 tumor tissues were isolated using percoll solution (17089109, Cytiva). The cells were re‐suspended supplemented with RPMI‐1640 medium with 10% FBS and added leukocyte activation cocktail with BD GolgiPlug (550583, BD), placed then culture in a 37 °C CO2 incubator for 6 h. Following activation, the cells were harvested and washed with 1X PBS. Before staining, the cells were dyed with Fixable Viability Stain 780 (565388, BD Pharmingen) to distinguish viable cells and blocked with Ultra‐LEAF Purifed α‐mouse CD16/32 (101339, BioLegend). The immune cells isolated from 4T1 tumor tissues suspensions were fluorescently stained with the following detection antibodies: α‐CD45 (553079, BD), α‐CD3ε (566494, BD), α‐CD4 (563151, BD), α‐CD8α (557959, BD), α‐CD49b (740895, BD), α‐Ki67 (563462, BD), α‐CD69 (552879, BD), α‐CD25 (557192, BD), α‐Granzyme‐B (130‐116‐486, Miltenyi Biotec), α‐IFN‐γ (563854, BD), α‐CXCR3 (562152, BD), α‐CD11b (563015, BD), α‐Gr‐1 (552093, BD), α‐CD11c (557401, BD), α‐MHC‐II (743871, BD), α‐F4/80 (565411, BD), α‐CD86 (560582, BD), α‐CD206 (568808, BD). The cell suspensions of lung tissues were fluorescently stained with the following detection antibodies: α‐CD45 (103132, BioLegend), α‐CD3ε (100206, BioLegend), α‐CD8α (100706, BioLegend), α‐CD49b (108923, BioLegend), α‐Ki67 (151215, BioLegend), α‐CD69 (104536, BioLegend), α‐CD25 (102012, BioLegend), α‐Granzyme‐B (372214, BioLegend), α‐IFN‐γ (505838, BioLegend), α‐CD11b (101205, BioLegend), and α‐Gr‐1 (108421, BioLegend). Antibody staining of cell suspensions for flow cytometry analysis was performed following the antibody manufacturer's recommendations. The auxiliary reagents used in this assay were BD Pharmingen Stain Buffer (554658) and True‐Nuclear Transcription Factor Buffer Set (424401, BioLegend). Flow cytometry was performed using Beckman CytoFLEX LX, and FACS data were analyzed by FlowJo_V10.

### IHC and IF Staining

Freshly isolated 4T1 tumors or lung tissues were fixed in a 10% formalin solution for 48 h. The fixed tissues were then dehydrated, paraffin‐embedded, sectioned, and transferred to slides. Anti‐TGF‐β1 (ab215715, Abcam), anti‐VEGFA (ab1316, Abcam), anti‐E‐cadherin (3195, CST), anti‐Vimentin (5741, CST), and anti‐N‐cadherin (ab18203, Abcam) IHC staining assays were performed following a two‐step protocol. Bright‐field images were captured by the Nanozoomer slide‐scanning platform. IF staining based on tyramine signal amplification was performed according to the manufacturer's recommendations. anti‐α‐SMA (19245, CST), anti‐CD31 (ab28364, Abcam)), anti‐CD3 (ab16669, Abcam), anti‐CD4 (ab183685, Abcam), and anti‐CD8 (98941, CST) were used in the IF assay. Images of IF were captured by fluorescence microscopy, and previewed via SlideViewer software, and ROIs were defined by two experienced pathologists. The quantitative analysis of IHC and IF images was conducted with ImageJ software.

### RNA Sequencing (RNA‐seq) Assay

4T1 tumor tissues were harvested on day 7. Total RNA extraction using Total RNA extraction Kit (DP419, TIANGEN). The quality and quantity of the purified RNA were determined by measuring the absorbance at 260nm/280nm(A260/A280) using an Ultrafine spectrophotometer (N50 touch, IMPLEN). The integrity of the RNA was further verified by 1.0% agarose gel electrophoresis. RNA‐seq was conducted on the Illumina Novaseq 6000 platform (performed by Wuhan RuiXing) The reference genome utilized was Mus_musculus GRCm39, version M27 (Ensembl 104). DEGs between different groups were screened with the DESeq2 package in R software, with criteria set for fold change (FC) ≥ 2 or ≤ 0.5 and false discovery rate (FDR) < 0.05. Gene signatures were based on published lists.^[^ ^73^, ^74^ ^]^ Signature scores were defined as the mean Z‐score among all genes in each gene signature.

### Statistical Analysis

Statistical analyses were conducted by R software and GraphPad Prism V. 8 software. Student's *t*‐test with or without Welch's correction and Mann–Whitney test was applied to compare two groups. Mouse Kaplan–Meier survival curves were compared by the log‐rank test. Data were presented as mean ± SE of mean (SEM) or SD. All tests in this study were two‐sided and *P*<0.05 indicated a significant difference.

### Ethics Approval and Consent to Participate

The animal operations in this study were evaluated and approved by the Institutional Animal Care and Use Committee of The Second Affiliated Hospital of Xi'an Jiaotong University (No. 202210215).

## Conflict of Interest

J.Z., L.Z., Y.X., H.M.W., and P.F.Z. were employees of Wuhan YZY Biopharma Co., Ltd.

## Author Contributions

L.J.L. conducted the experiments, analyzed data, prepared the figures, and drafted the manuscript. M.Y. revised the manuscript and the figures and provided suggestions for the manuscript. J. C., L. Z., Y. X., HM. W., and YJ. D. participated in animal experiments and/or provided technical assistance. X.B.M., J.Z., X.J.Z., and P.F.Z. provided technical and/or material support and participated in the analysis and interpretation of data. K.M.W., H.F. K., and Z.J.D. designed the work and supervised the study. All authors gave final approval of the version to be published and agreed to be accountable for all aspects of the work.

## Supporting information



Supporting Information

## Data Availability

The data that support the findings of this study are available from the corresponding author upon reasonable request.
