# Supplementary material for: Bispecific Antibody Targeting VEGF/TGF‐β Synergizes with Local Radiotherapy: Turning Tumors from Cold to Inflamed and Amplifying Abscopal Effects
Source: Adv Sci (Weinh). 2025 Jun 5;12(30):e01819. doi: 10.1002/advs.202501819 (PMC12376579; doi:10.1002/advs.202501819)
Supplement: Supplementary file 1 — Supporting Information [file ADVS-12-e01819-s001.doc]

Supporting Information

**Bispecific antibody targeting VEGF/TGF-β synergizes with local radiotherapy: Turning tumors from cold to inflamed and amplifying** **abscopal effects**

Lijuan Lyu^1^, Ming Yi^2^, Ji Chen^3^, Jing Zhang^4^, Xiaobin Ma^1^, Xiaojun Zhang^5^, Liang Zeng^4^, Yan Xue^4^, Haimei Wen^4^, Yujiao Deng^1^, Pengfei Zhou^4^, Kongming Wu^5,6*^, Huafeng Kang^1*^, and Zhijun Dai^1,2*^


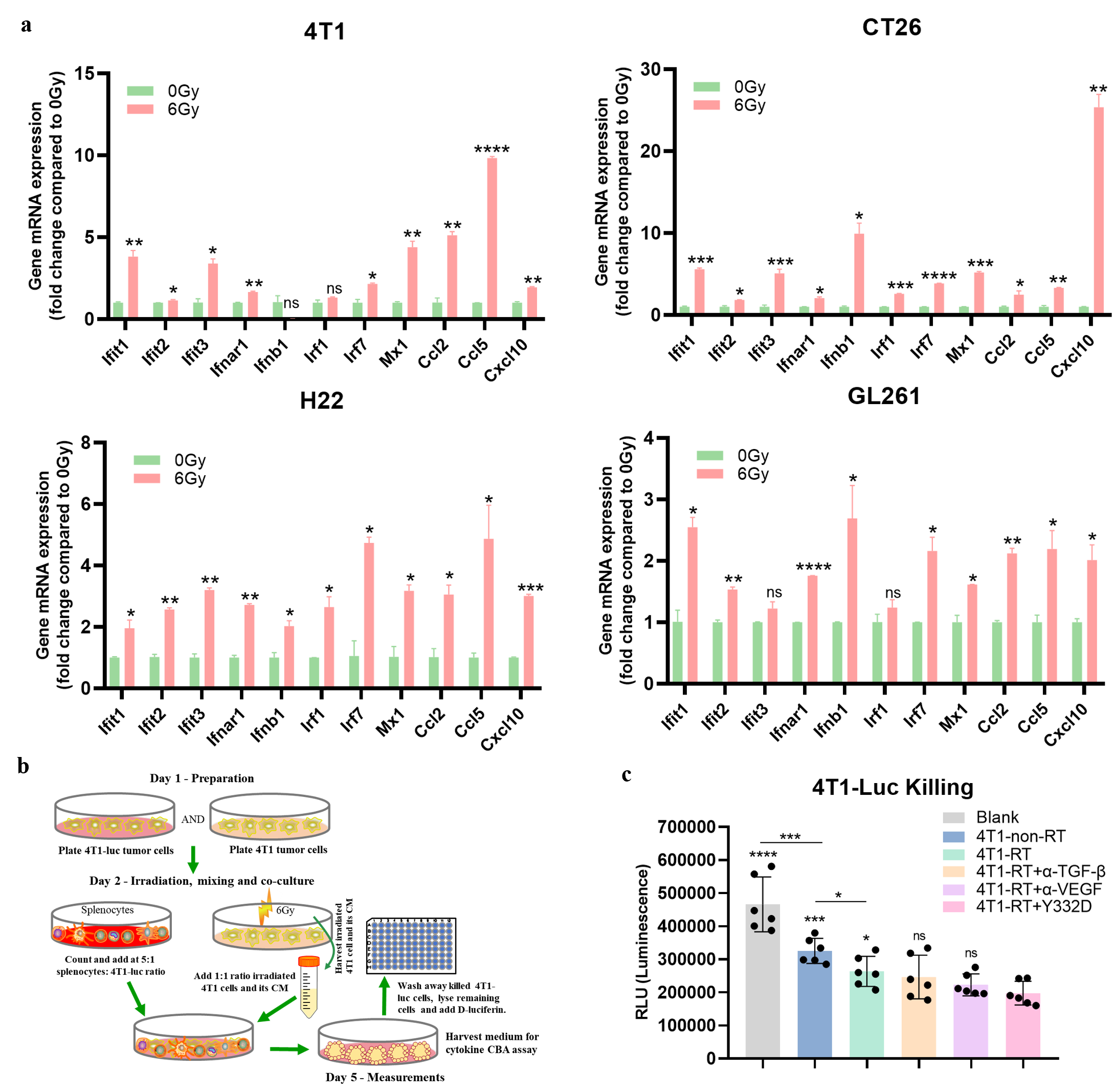


**Figure S1.** a) The mRNA expression of a panel of 11 IFN-stimulated genes (ISGs) in 4T1, CT26, H22, and GL261 cells 24 hours post-irradiation and non-irradiation was verified by qRT-PCR (n = 3). b) Graphical abstract illustrates the co-culture assay setup involving 4T1-Fluc tumor cells and effector splenocytes. The antibodies (10^6^ pM) were added to the co-culture system on day 2, and on day 5, the supernatants were harvested to measure cytokines concentration. c) The efficacy of effector cells in killing target 4T1-Fluc tumor cells was quantified on day 3 post-co-culture initiation. Luminescence units (RLU) were used to measure the remaining viable 4T1-Fluc cells (n = 6). All data are mean±SD. Statistical analyses were conducted using Student’s t-test. Statistical significance is denoted as follows: *p<0.05, **p<0.01, ***p<0.001, and ****p<0.0001; ns: not significant.


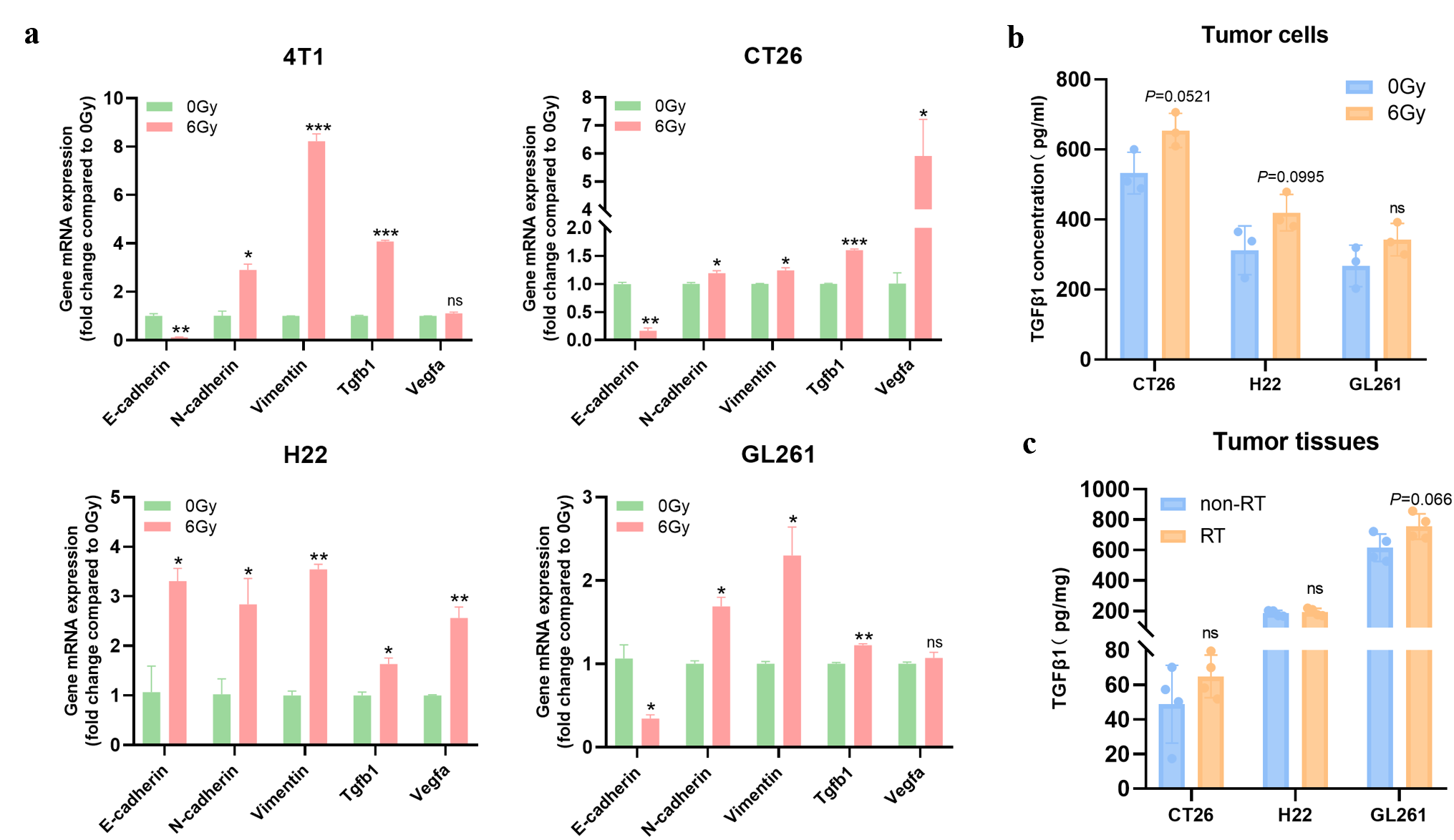


**Figure S2.** a) The mRNA expression of TGF-β1, VEGFA, and EMT-related genes in 4T1, CT26, H22, and GL261 cells 24 hours post-irradiation and non-irradiation was verified by qRT-PCR (n = 3). b) The concentration of TGF-β1 in the supernatants of CT26, H22, and GL261 cells was measured 48 hours after irradiation or non-irradiation by ELISA (n = 3). c) The TGF-β1 levels in tumor tissue homogenates of CT26, H22, and GL261 tumors at 3 days after RT (6 Gy*3) quantified by ELISA (n = 4). All data are mean±SD. Statistical analyses were conducted using Student’s t-test. Statistical significance is denoted as follows: *p<0.05, **p<0.01, ***p<0.001, and ****p<0.0001; ns: not significant.


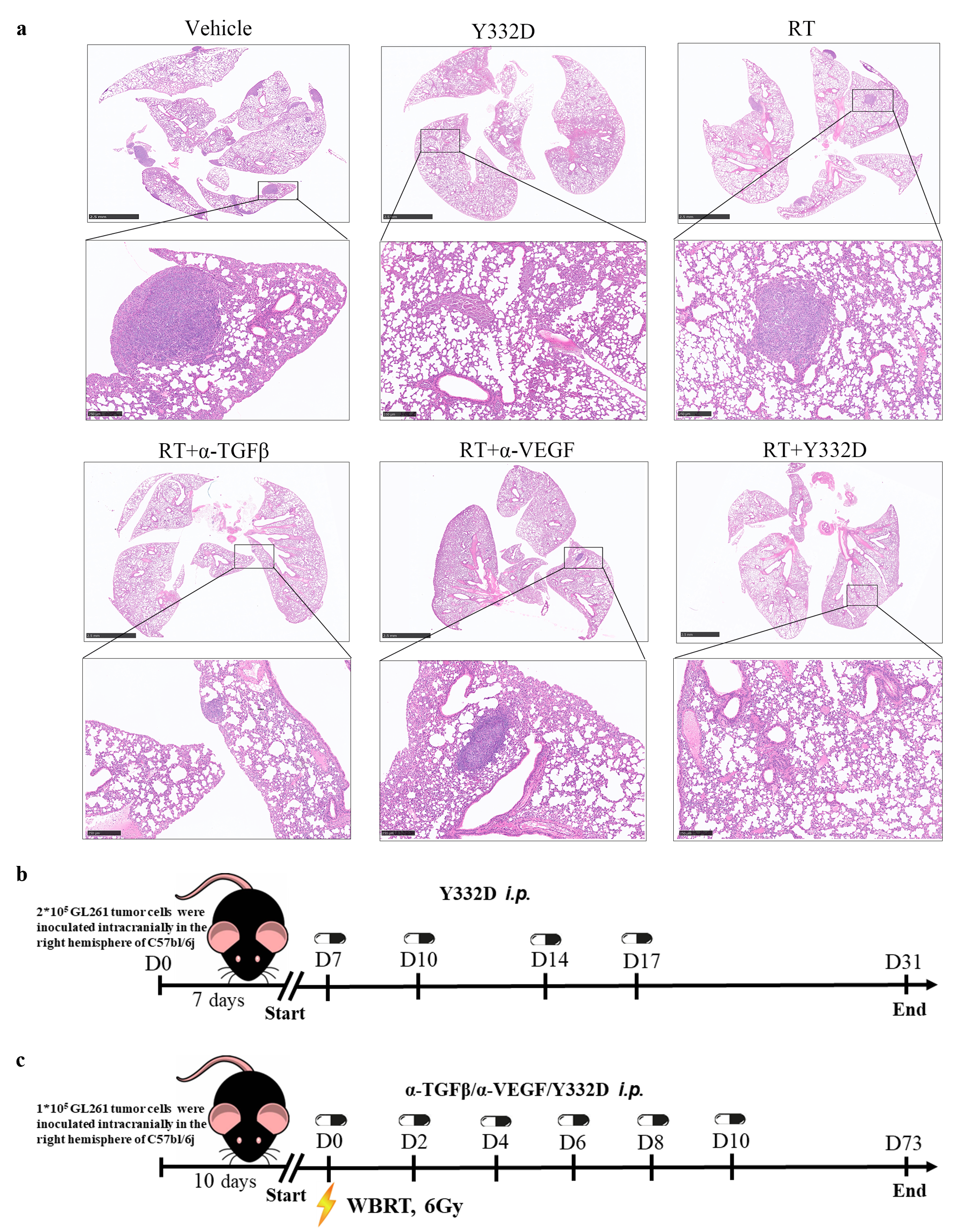


**Figure S3.** a) The representative H&E staining images of lung tissues from mice bearing 4T1 orthotopic tumors that received combination or monotherapy treatments. The scale bar in H&E images is 2.5 mm or 250 μm. b) Schematic representation of the treatment schedule for the orthotopic GL261 glioblastoma model receiving Y332D monotherapy. c) Schematic representation of the treatment schedules for the orthotopic GL261 glioblastoma model receiving combination therapies or monotherapies. WBRT: ole brain radiotherapy.


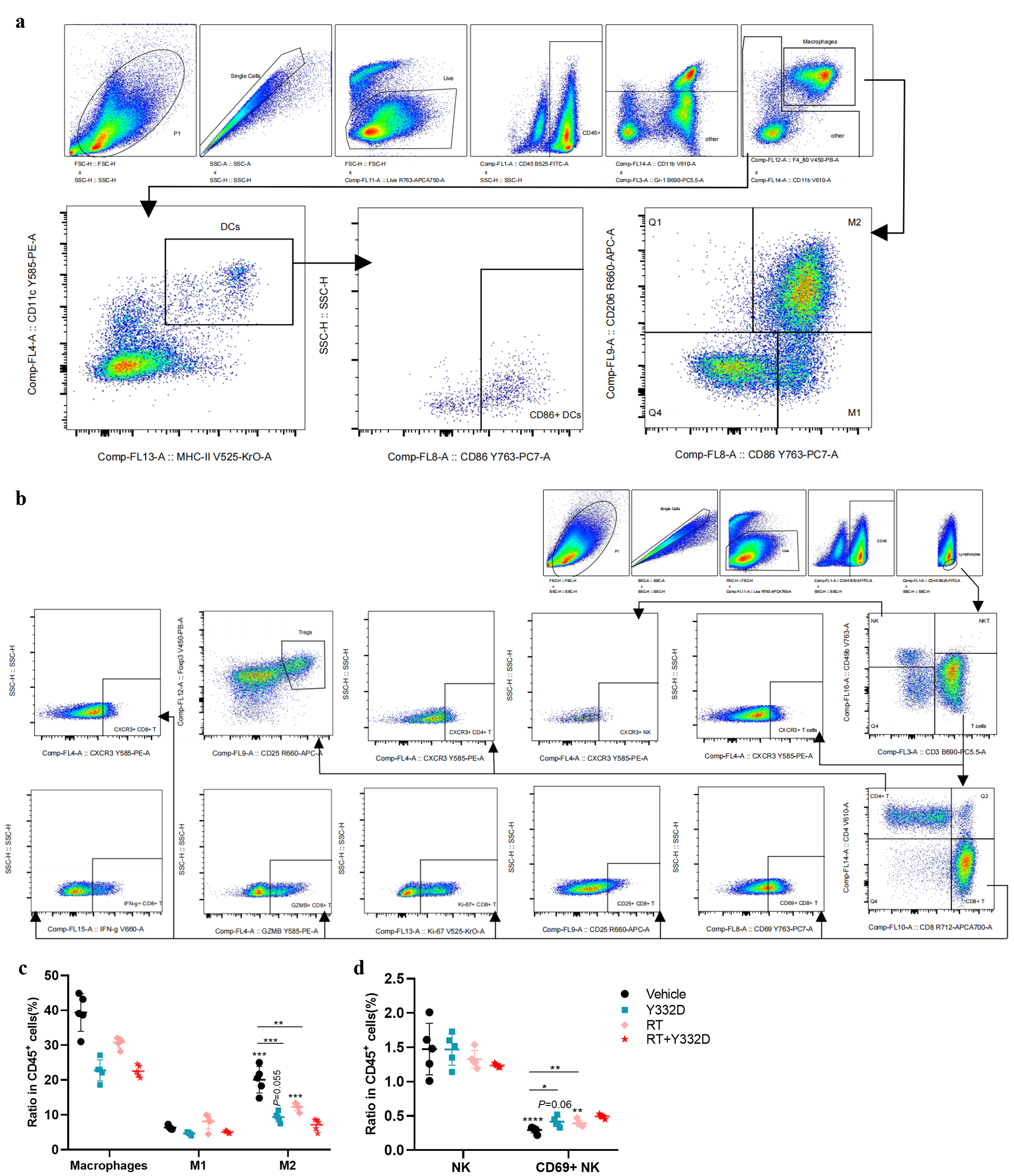


**Figure S4.** Flow cytometry assays to analyze tumor-infiltrating immune cells in 4T1 tumor model. a, b) Flow cytometry gating strategies for identifying tumor-infiltrating T, NK, DC, and macrophages. c)The percentage of macrophages, M1-type macrophages, and M2-type macrophages in CD45+ cells intratumor assessed by flow cytometry assays (n = 5). d) The percentage of NK cells and CD69+ NK cells in CD45+ cells intratumor assessed by flow cytometry assays (n = 5). All data are mean±SD. Statistical analyses were conducted using Student’s t-test. Statistical significance compared to RT combined with Y332D is indicated as follows: *p<0.05, **p<0.01, ***p<0.001, and ****p<0.0001; ns: not significant.


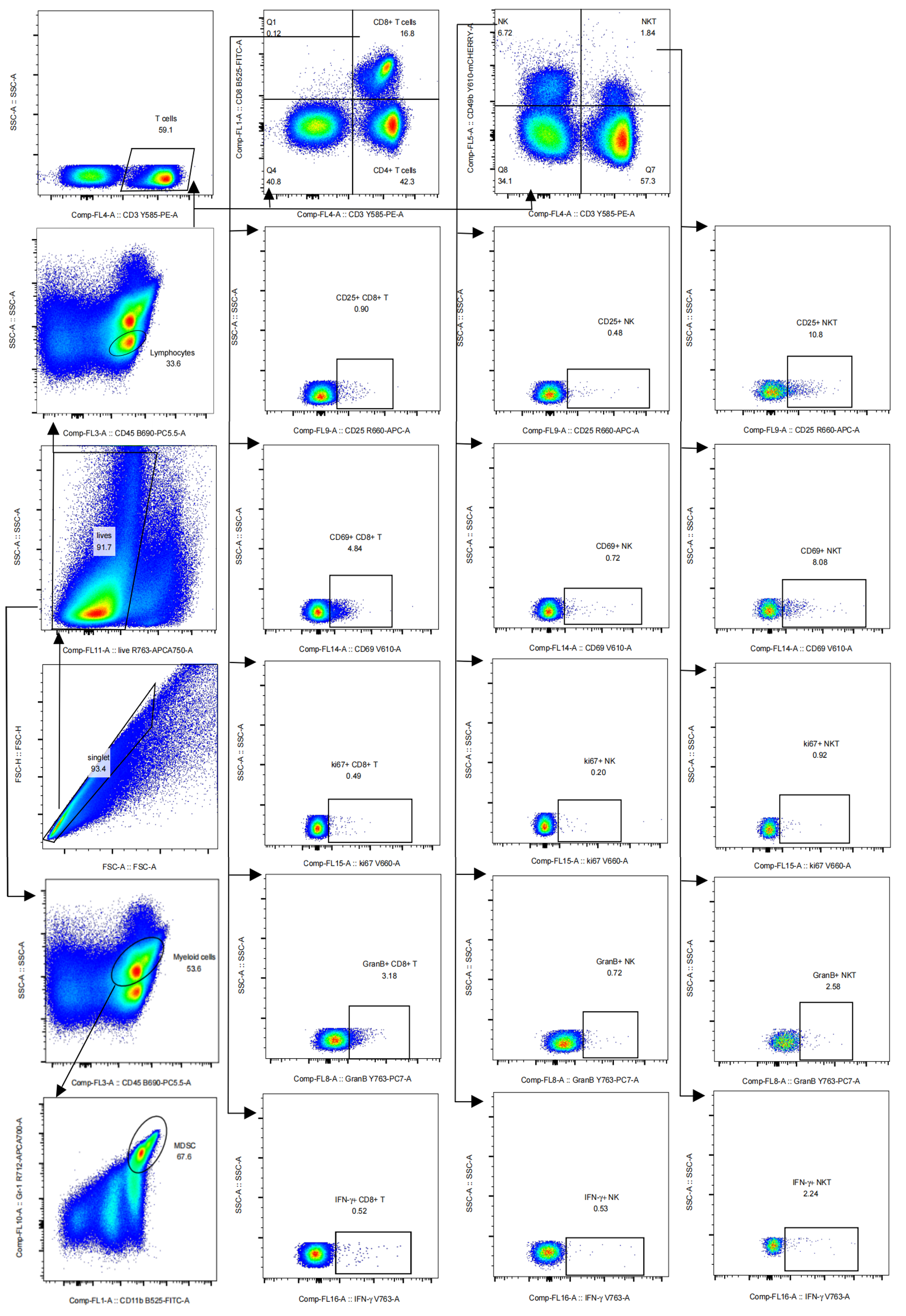


**Figure S5**. Flow cytometry gating strategies for identifying T cell, NK cell, NKT cells, and MDSCs in lung tissue of mouse bearing 4T1 orthotopic tumor.

| Gene name | Forward | Reverse |
| --- | --- | --- |
| Gapdh | 5′-CGACTTCAACAGCAACTCCCACTCTTCC-3′ | 5′-TGGGTGGTCCAGGGTTTCTTACTCCTT-3′ |
| Ifnb1 | 5′-GCCTTTGCCATCCAAGAGATGC-3′ | 5′-ACACTGTCTGCTGGTGGAGTTC-3′ |
| Ifnar1 | 5′- CCAAGGCAAGAGCTATGTCCTG-3′ | 5′-CAGTGCGTAGTCTGGACATTTGC-3′ |
| Mx1 | 5′-TGGACATTGCTACCACAGAGGC-3′ | 5′-TTGCCTTCAGCACCTCTGTCCA-3′ |
| Irf1 | 5′-TCCAAGTCCAGCCGAGACACTA-3′ | 5′-ACTGCTGTGGTCATCAGGTAGG-3′ |
| Irf7 | 5′-CCTCTGCTTTCTAGTGATGCCG-3′ | 5′-CGTAAACACGGTCTTGCTCCTG-3′ |
| Ifit1 | 5′-TACAGGCTGGAGTGTGCTGAGA-3′ | 5′-CTCCACTTTCAGAGCCTTCGCA-3′ |
| Ifit2 | 5′-CGAACTACCGTCTGGATGACTG-3′ | 5′-CTTCAACCAGCGCCATTGCTTG-3′ |
| Ifit3 | 5′-GCTCAGGCTTACGTTGACAAGG-3′ | 5′-CTTTAGGCGTGTCCATCCTTCC-3′ |
| Ccl2 | 5′-GCTACAAGAGGATCACCAGCAG-3′ | 5′-GTCTGGACCCATTCCTTCTTGG-3′ |
| Ccl5 | 5′-CCTGCTGCTTTGCCTACCTCTC-3′ | 5′-ACACACTTGGCGGTTCCTTCGA-3′ |
| Cxcl10 | 5′-ATCATCCCTGCGAGCCTATCCT-3′ | 5′-GACCTTTTTTGGCTAAACGCTTTC-3′ |
| Tgfb1 | 5′-TGATACGCCTGAGTGGCTGTCT-3′ | 5′-CACAAGAGCAGTGAGCGCTGAA-3′ |
| Vefga | 5′-CTGCTGTAACGATGAAGCCCTG-3′ | 5′-GCTGTAGGAAGCTCATCTCTCC-3′ |
| E-cadherin | 5′-TCATGAGTGTCCCCCGGTAT-3′ | 5′-TCTTGAAGCGATTGCCCCAT-3′ |
| N-cadherin | 5′-CCTCCAGAGTTTACTGCCATGAC-3′ | 5′-CCTCCAGAGTTTACTGCCATGAC-3′ |
| Vimentin | 5′-GGACCAGCTAACCAACGACA-3′ | 5′-AAGGTCAAGACGTGCCAGAG-3′ |

**Table S1.** qRT-PCR primer sequences used in this study.
